# A bacteriophage mimic of the bacterial nucleoid-associated protein Fis

**DOI:** 10.1042/BCJ20200146

**Published:** 2020-04-17

**Authors:** Soumyananda Chakraborti, Dhanasekaran Balakrishnan, Alexander J. Trotter, William H. Gittens, Ally W.H. Yang, Arttu Jolma, Joy R. Paterson, Sylwia Świątek, Jacek Plewka, Fiona A. Curtis, Laura Y. Bowers, Lars-Olof Pålsson, Timothy R. Hughes, Michał Taube, Maciej Kozak, Jonathan G. Heddle, Gary J. Sharples

**Affiliations:** 1 Malopolska Centre of Biotechnology, Jagiellonian University, Krakow, Poland; 2 Postgraduate School of Molecular Medicine, Zwirki i Wigury 61, 02-091 Warsaw, Poland; 3 Department of Biosciences, Durham University, Stockton Road, Durham DH1 3LE, U.K.; 4 Department of Chemistry, Durham University, Stockton Road, Durham DH1 3LE, U.K.; 5 Donnelly Centre, University of Toronto, 160 College Street, Toronto, ON M5S 3E1, Canada; 6 Department of Macromolecular Physics, Faculty of Physics, Adam Mickiewicz University, Uniwersytetu Poznańskiego 2, PL 61-614 Poznań, Poland; 7 National Synchrotron Radiation Centre SOLARIS, Jagiellonian University, Czerwone Maki 98, 30-392 Kraków, Poland

**Keywords:** bacteriophage λ, DNA bending, DNA binding, Fis, NinH, nucleoid-associated proteins

## Abstract

We report the identification and characterization of a bacteriophage λ-encoded protein, NinH. Sequence homology suggests similarity between NinH and Fis, a bacterial nucleoid-associated protein (NAP) involved in numerous DNA topology manipulations, including chromosome condensation, transcriptional regulation and phage site-specific recombination. We find that NinH functions as a homodimer and is able to bind and bend double-stranded DNA *in vitro.* Furthermore, NinH shows a preference for a 15 bp signature sequence related to the degenerate consensus favored by Fis. Structural studies reinforced the proposed similarity to Fis and supported the identification of residues involved in DNA binding which were demonstrated experimentally. Overexpression of NinH proved toxic and this correlated with its capacity to associate with DNA. NinH is the first example of a phage-encoded Fis-like NAP that likely influences phage excision-integration reactions or bacterial gene expression.

## Introduction

Efficient packaging is essential to accommodate the genome within a cell or cellular compartment while at the same time allowing access for the replicative, transcriptional and regulatory machinery. In bacteria, a group of histone-like proteins (e.g. Fis, IHF, H-NS and HU), collectively termed nucleoid-associated proteins (NAPs), fulfill a vital role in chromosome organization and gene expression [1,2]. Each of the NAPs perform discrete roles in intra- and intermolecular nucleoprotein assemblies and are often temporally regulated to suit the requirements of the cell cycle. The diverse functions of Fis include transcriptional regulation of a large number of genes at various stages of growth [3], including those involved in virulence [4].

Fis was originally identified due to its involvement in the topology manipulations necessary to drive site-specific inversion reactions involving the Hin and Gin recombinases [5]. Similarly, Fis works with Xis to direct excision of the λ prophage from the host chromosome [6–8] and can also stimulate phage integration reactions [9–11]. Fis condenses DNA by binding 21 bp segments via its non-specific DNA binding and bending activities [12]. However, Fis is also capable of forming stable complexes with multiple, specific DNA sequences found in regulatory areas of the genome [3]. High-affinity Fis binding sites display a degenerate consensus with a G/C dyad separated by a central AT-rich core of 13 bp [13–15]. Fis appears to initially select a DNA target based upon the narrow minor groove found at AT-rich sites, followed by sequence-specific contacts with the adjacent major grooves [14]. The Fis homodimer maintains its structural integrity while it draws the duplex over its two helix-turn-helix (HTH) motifs, stabilizing the association and inducing the DNA to bend by ∼65° [14,16,17]. Each HTH is held in place by multiple side-chain contacts with the phosphodiester backbone stabilized by interactions with selected bases in the major groove [14].

In this study, we identify the previously uncharacterized phage λ NinH protein as a Fis-like homolog. The *ninH* gene is located immediately downstream of *rap* (*ninG*) as part of the *ninR* region situated between the genes for replication proteins O and P and the Q antiterminator and adjacent lysis cassette [18]. We demonstrate that NinH has double-stranded DNA binding and bending activities that resemble those found with Fis. The expression of NinH confers significant negative effects on bacterial growth and this was used to monitor DNA binding *in vivo*. The results are consistent with NinH affecting both cellular and viral DNA processing and also gene expression, with potentially advantageous effects on phage multiplication and dissemination.

## Materials and methods

### NinH cloning

The *ninH* gene was amplified by PCR from λ genomic DNA (*c*I857 *S*am7) with oligonucleotides 5′-AAGTGAGGCCATATGACGTTCTCAG-3′ and 5′-AAATATTTCGGTATAAGCTTCCATC-3′. PCR products were inserted into pT7-7 (pFC109) and pET14b (pFC110) following digestion with NdeI and HindIII (underlined) to generate overexpression constructs of native NinH and N-His_6_-NinH (His-NinH), respectively. Nucleotide sequencing of these constructs revealed a guanine to adenine change at position 43 of the coding sequence relative to the published genome sequence of λ, although it matches that derived from λ *c*I857 *S*am7. The alteration produces an alanine to threonine substitution at position 15 of NinH; both amino acids are represented among phage NinH family members at this position. Synthetic genes encoding His-NinH and site-directed mutants with flanking NdeI and XhoI restriction enzyme sites inserted into pET28a(+) were purchased from Twist Bioscience (San Francisco). The sequence of this codon-optimized synthetic gene and its mutant derivatives are listed in Supplementary Figure S1A. The *ninH* gene was synthesized and inserted into pTH6838 [19] by Genscript to generate an N-terminal GST-NinH fusion. The sequence of this insert is shown in Supplementary Figure S1B.

### Purification of NinH

*Escherichia coli* BL21-AI (Invitrogen; F^−^ *ompT gal dcm lon hsdS*_B_(*r*_B_^−^ *m*_B_^−^) *araB*::*T7RNAP*-*tetA*) cells carrying pFC109 (native NinH; 2 l) or pFC110 (His-NinH; 500 ml) were grown in LB broth supplemented with 150 µg/ml ampicillin. NinH expression was induced with 0.5 mM IPTG and 0.2% (w/v) l-arabinose when the culture reached an A_650nm_ of 0.5 and incubation continued for 3 h at 37°C. For NinH, harvested cells were resuspended in 20 mM Tris–HCl pH8, 1 mM EDTA, 0.5 mM DTT (Buffer A), lysed by sonication and the cleared lysate applied to a 10 ml Q-sepharose fast flow column (GE Healthcare); the majority of the NinH protein appeared in the flow through and wash fractions. The combined flow through and wash samples containing NinH were loaded onto a 3 ml dsDNA cellulose column (Sigma) and bound proteins eluted in Buffer A with a gradient of 0.2–1 M KCl. NinH eluted between 0.35 and 0.5 M KCl and pooled fractions were applied to a 1 ml heparin agarose column (Sigma). NinH eluted from the column between 0.4 and 0.8 M KCl and peak fractions were dialyzed in Buffer A containing 50% (v/v) glycerol and stored in aliquots at −80°C.

Cells containing overexpressed His-NinH were resuspended in 20 ml 50 mM NaH_2_PO_4_ pH 8, 300 mM NaCl, 10 mM imidazole and lysed by sonication. The cleared lysate was applied to a 1.5 ml His-Select^™^ Nickel affinity gel (Sigma) column and bound proteins eluted in buffer containing 250 mM imidazole. His-NinH subsequently precipitated, eliminating some additional minor contaminants, and was resuspended in 20 mM Tris–HCl pH8, 1 mM EDTA, 0.5 mM DTT, 1 M KCl. Purified protein was dialyzed against 20 mM Tris–HCl pH8, 1 mM EDTA, 0.5 mM DTT, 0.5 M KCl, 50% (v/v) glycerol and stored in aliquots at −80°C. Additional, His-NinH and mutant proteins were isolated from *E. coli* BL21-Star (Invitrogen; F^−^ *ompT gal dcm lon hsdS*_B_(*r*_B_^−^ *m*_B_^−^) *rne131* (DE3)) cells carrying pET14b and pET28a plasmid constructs. Cells were grown in LB broth supplemented with 100 µg/ml ampicillin and induced with 1 mM IPTG at an A_600nm_ of 0.6 and incubation continued for 4 h at 37°C. Harvested cells were resuspended in 20 ml column buffer (50 mM NaH_2_PO_4_ pH8, 300 mM M NaCl, 1 mM EDTA) containing 10 mM imidazole and lysed by sonication. The cleared lysate was applied to a 5 ml His Trap column (GE Healthcare) and bound proteins eluted in buffer containing a 0–500 mM gradient of imidazole. Purified His-NinH was dialyzed (3 kDa cut-off) against the column buffer. Contaminating DNA was removed using a 5 ml HiTrap Heparin column (GE Healthcare), with bound His-NinH eluted using a gradient of 0.3–1.0 M NaCl. Amounts of NinH are expressed as moles of dimer.

### *In silico* NinH structure prediction

The structure of the full-length NinH dimer was validated by fitting an *in silico* model to data generated from small angle X-ray scattering (SAXS). The NinH monomer structure was homology-modeled using *E. coli* Fis as a template. The sequences of NinH (UniProt ID P03771) and Fis (UniProt ID P0A6R3) protein were aligned using Clustal Omega by creating two separate sequence alignments (aligning NinH residues 1–44 to the C-terminal region of Fis and NinH residues 45–69 to the N-terminal region of Fis). Two different templates were generated based on the same Fis protein. These templates were used as the input for building NinH monomer models. A multi-template modeling technique was implemented in Modeller9v21 [20] with the default parameters for building the monomer NinH models. A total of 125 NinH monomer models were built and the 15 best models shortlisted based on alignment to Fis. The criteria used for model selection were: (a) minimizing the RMSD of the structural alignment between the DNA binding region of predicted NinH monomer models residues 29–35 and Fis protein and (b) evaluation of the overall similarity of the C-terminal region between the predicted monomer models and Fis. The NinH homodimer was then modeled using the HADDOCK online web tool [21,22]. The monomer models shortlisted previously were used as inputs and two different sets of DNA binding or dimerization (either His45, His55, Arg56 or His55, Arg56) domain residues were used as constraints for docking the two monomers. A total of 1200 homodimer structures were generated from the 15 monomer models and RMSD was calculated between the residues in the DNA binding region of the predicted models and Fis. A final refinement step was carried out on the favored model using the GalaxyRefineComplex online web tool [23,24] where the model was relaxed and 10 refined models generated. All structures were validated for Ramachandran outliers using the RAMPAGE server [25] and the best model (98.5% of residues in the favored region) was chosen for SAXS validation.

### SAXS data collection and modeling

To determine the 3D structure of NinH protein under native conditions we performed SAXS analysis using a laboratory SAXS system Xeuss 2.0 (XENOCS, Grenoble, France) equipped with a MetalJet D2 microfocus X-ray generator (0.134 nm wavelength). The His-NinH solution at 4 mg/ml was centrifuged to remove any potential aggregates and injected into the low-noise liquid sample cell. SAXS data were collected in four sequential 10 min long frames using a Pilatus3R 1 M hybrid photon counting detector (Dectris, Switzerland). Data reduction and buffer subtraction procedures were performed using the Foxtrot package [26]. Resulting scattering profiles were checked for radiation damage and averaged. All structural parameters were calculated using Primus software from the ATSAS package [27]. The distance distribution function was calculated using GNOM [28]. The low-resolution molecular envelope was calculated using 10 rounds of DAMMIF [29] analysis followed by clustering and filtering. Resulting SAXS data were used to validate the quality and probability of the *in silico* model of the NinH dimer. Since SAXS measurements were performed using His-NinH dimer, flexible overhangs were added to the model in PyMOL to compensate for their electron density.

### Quaternary analysis

The oligomeric states of His-NinH wild-type and mutant proteins were estimated by analytical ultracentrifugation (AUC, Beckman) at a concentration of ∼0.6 mg/ml. Sedimentation velocity measurements were performed in a Beckman-Coulter ProteomeLab XL-I analytical ultracentrifuge, equipped with AN-50Ti rotor (8-holes) and 12 mm path length. Experiments were conducted at 4°C at 50 000 rpm in continuous scan mode. Scans were collected at 8 min intervals at both 260 and 280 nm. Fitting of absorbance versus cell radius data was carried out using SEDFIT software. Oligomer formation by His-NinH was also examined using boiled and un-boiled protein samples separated by 15% SDS–PAGE and visualized by staining with either InstantBlue^TM^ (Expedeon) or Coomassie blue. A pre-stained protein molecular mass standard (Bio-Rad or ThermoFisher) was used as a size marker.

### DNA binding assays

Oligonucleotides used to make the DNA substrates used in this study are listed in Supplementary Table S1. Electrophoretic mobility shift assays (20** **µl) using ^32^P-labeled DNA substrates were conducted in 50** **mM Tris–HCl pH 8.0, 5** **mM EDTA, 1** **mM dithiothreitol, 5% (v/v) glycerol, 100** **µg/ml BSA. Samples were incubated on ice for 15 min prior to separation on 4% (w/v) PAGE in 6.7** **mM Tris–HCl pH 8.0, 3.3** **mM sodium acetate, 2** **mM EDTA at 160** **V. Gels were dried on filter paper and analyzed by autoradiography and phosphorimaging. Additional gel shift assays were performed in PBS (137** **mM NaCl, 2.7** **mM KCl, 8** **mM Na_2_HPO_4_ and 2** **mM KH_2_PO_4_ pH 7.4) with His-NinH and mutant derivatives incubated with Cy5-labeled dsDNA (Cy5_20_) for 30 min at room temperature. In all cases, the DNA concentration was kept constant at 10** **nM while protein concentrations varied. DNA–protein mixtures were separated by electrophoresis on 7% native PAGE (6.7** **mM Tris–HCl pH 8.0, 3.3** **mM sodium acetate, 2** **mM EDTA) at 150** **V for 35 min. Gels were scanned using a Typhoon scanner (GE Healthcare) and images analyzed and quantified by Image Quant software (GE Healthcare).

Steady-state fluorescence anisotropy measurements were taken at 25°C on a Carey Eclipse Spectrophotometer (Agilent Technologies). A sample (900** **µl) containing fluorescein-labeled 10** **nM dsDNA (o-FLU_20_ annealed to o-SS_20_) in 6.7** **mM Tris–HCl pH 8.0, 3.3** **nM sodium acetate, 2** **mM EDTA was added to a 1** **ml quartz cuvette. Fluorescein anisotropy was determined from λ^em^ (520** **nm) with λ^ex^ (495** **nm). Samples were excited with a PMT voltage of 850** **V and the excitation and emission slits at 5** **nm. The G-factor was measured at the outset and each anisotropy titration calculated using WinFLR software (Agilent Technologies).

Microscale thermophoresis (MST) was also employed to monitor binding to the Cy5_20_-labeled dsDNA substrate in PBS with 0.05% (v/v) Tween-20 (PBST). Proteins were serially diluted (0.03–1000 nM) in PBST buffer in PCR tubes. The Cy5-labeled DNA (2.5–5 nM) was incubated with His-NinH for 30 min at RT, prior to measurement. After incubation, samples were introduced to premium capillaries (Catalogue # MO-K025, Monolith) and run on a Monolith NT.115. Sample measurement was carried out at 22°C and the determination of *K*_D_ values was performed using proprietary software (Nanotemper).

GST-NinH produced *in vitro* using T7 RNA polymerase from the *ninH* gene cloned in pTH6838 was employed in a protein binding microarray (PBM) assay as described previously [30]. Preferential sequence binding with the fusion protein was performed in duplicate on two microarrays with differing probe sequences (denoted ME and HK; [31] in PBS, 2% (w/v) skimmed milk, 0.2 mg/ml BSA, 50 μM zinc acetate, 0.1% Tween-20; 8-mer *Z* and *E* scores were calculated as described by Berger *et al*. [30]. GST-NinH protein was also tested with a bead-based HT-SELEX (high-throughput-systematic evolution of ligands by exponential enrichment) method essentially as described [32,33]. The SELEX process was repeated three times in 140 mM KCl, 5 mM NaCl, 1 mM K_2_HPO_4_, 2 mM MgSO_4_, 100 µM EGTA, 1 mM ZnSO_4_, 20 mM HEPES-HCl pH 7 and aliquots of the enriched DNA were sequenced at each cycle.

### DNA bending assays

Steady-state Förster resonance energy transfer (ssFRET) and time-resolved Förster resonance energy transfer (trFRET) were used to estimate the amount of DNA bending. Steady-state FRET was measured using a Perkin–Elmer LS 55 Fluorescence Spectrometer at room temperature in a final volume of 50 µl with 50 nM DNA. Assays used 1 μM protein and were incubated for 10 min at room temperature prior to analysis. Donor (fluorescein) was excited at 494 nm and emission spectra collected from 490 to 650 nm. Acceptor (tetramethylrhodamine) was directly excited at 558 nm and emission spectra collected from 558 to 650 nm. Excitation and emission bandwidths were 5 nm. Spectra were collected for both donor and donor–acceptor labeled dsDNA and FRET calculated from the increase in acceptor emission using the ratio_A_ method [34]. Ratio_A_ was used to calculate energy transfer efficiency (*E*) using the equation *E *= (*ε_A_*[558]/ = *ε_D_*[494]) × ratio_A_ − (*ε_A_*[494]/ = *ε_A_*[558]) where *ε_A_*[λ] and *ε_D_*[λ] are the acceptor and donor extinction coefficients provided by the supplier. Dye–dye distances (*R*) were calculated using the equation *E *= *R*_0_^6^/(*R*_0_^6^+*R*^6^) [34]. A Förster distance (*R*_0_) of 50 Å was used in agreement with previous studies [35,36].

The trFRET was assessed using the time-correlated, single-photon counting technique (TCSPC). Oligonucleotides and sample preparation were as for steady-state FRET. Time-resolved fluorescence spectroscopy was performed using a PicoQuant pulsed diode laser LDH-P-C-485 (485 nm, 70 ps pulses full width half maximum (FWHM) at 20 MHz). The emission was detected in a right-angle geometry using suitable band pass/long pass filters (Comar Instruments) with a photon counting module Idquantic (id100-20) in combination with a Becker–Hickl SPC-130 time-correlated single-photon counting module. The data were subsequently fitted to a sum of exponentials; $F(\lambda ,t)=\sum_{i}A_{i}\exp(-k_{i}t)$, by deconvolution with the instrument response function (IRF) obtained from a light scattering solution of Ludox particles. The IRF has a FWHM of ∼250 ps which provides a temporal time-resolution of ≥50 ps in the time-resolved fluorescence spectroscopy experiments. Fluorescence decays were collected for both donor and donor–acceptor labeled dsDNA with or without protein. Data were analyzed by the Grinvald–Steinberg method [37] to obtain the fluorescence lifetime for the donor and acceptor (*τ_DA_*) and donor only (*τ_D_*) labeled oligonucleotides. Energy transfer efficiency was calculated using the equation $E=1-\frac{\tau_{DA}}{\tau_{D}}$.

### Bacterial growth assays

BL21-AI cells transformed with plasmids carrying the *ninH* wild-type gene and substitution mutations and the relevant vector controls, pET28a(+) or pET14b, were cultivated at 37°C in LB broth supplemented with 50 µg/ml kanamycin or 200 µg/ml ampicillin to an A_650nm_ of 0.4. Ten-fold serial dilutions were performed in cold LB broth and 10 µl aliquots spotted onto agar plates containing antibiotic, arabinose (0.2%) and IPTG (1 mM) as appropriate. Plates were incubated at 30°C for 24–36 h and the extent of growth visualized.

## Results

### NinH resembles Fis

**Figure 1.**
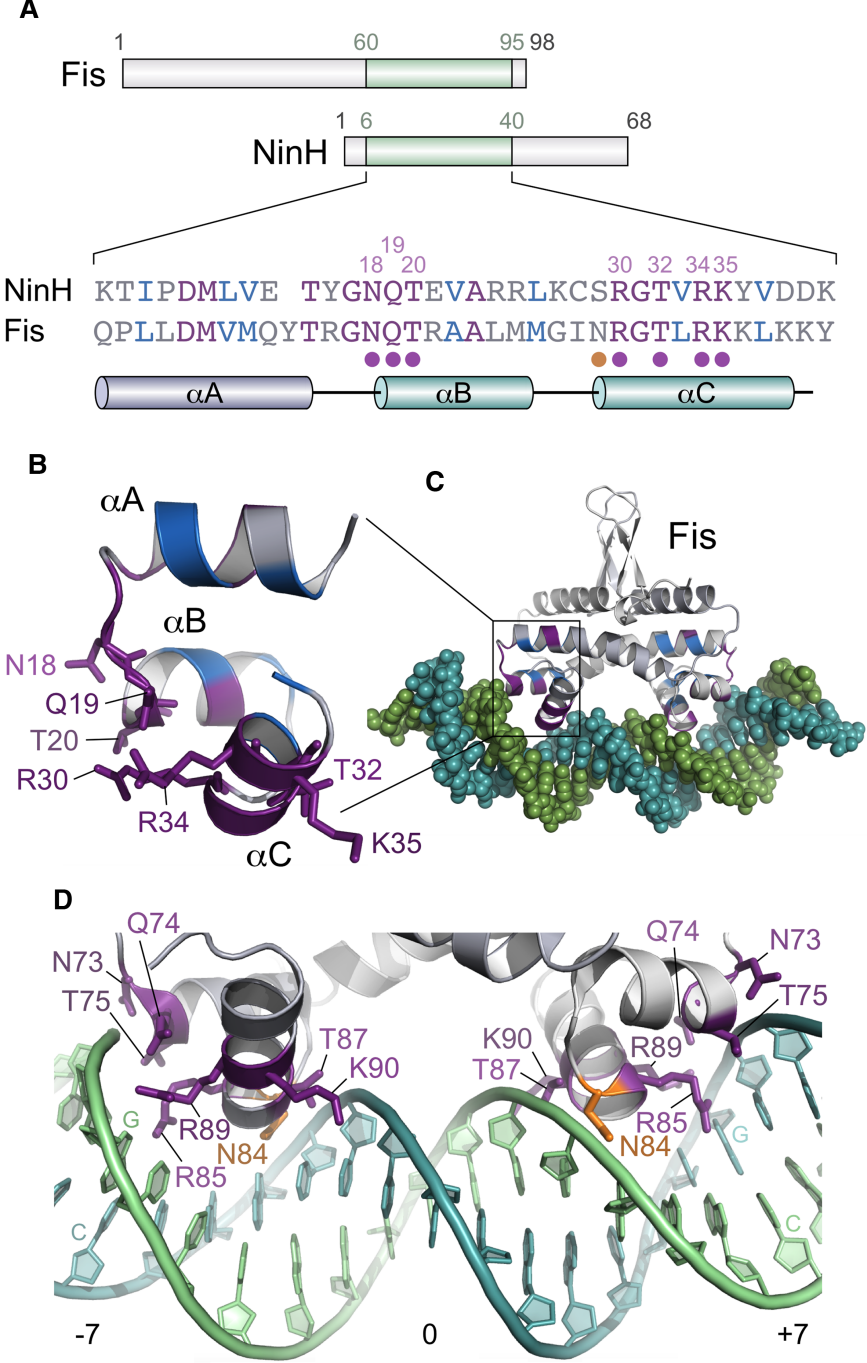
Sequence and structural similarities between NinH and Fis. (**A**) Sequence homology between NinH and the Fis DNA binding domain. The position of homologous regions is depicted on the 98-residue Fis and 68-residue NinH proteins. The sequence alignment highlights identical residues (purple), functionally equivalent amino acids (blue) and others (gray). Residues in the Fis-DNA structure that participate in DNA binding are indicated by a filled circle. With the exception of Asn84 (orange circle), all of these are conserved in λ NinH and labeled above the primary sequence. Helices are labeled αA, αB and αC in NinH and correspond to helices αB, αC and αD in Fis, the latter two helices comprise the helix-turn-helix. (**B**) The NinH structure modeled by Phyre^2^ [42] on the *apo* Fis structure (PDB 1FIP). Residues are colored as in (**A)** and those matching the key residues in Fis that contact DNA are shown in stick format and labeled. (**C**) Crystal structure of the Fis–DNA complex [14]; PDB 3IV5) also showing residues conserved between NinH and Fis. (**D**) Detail of the Fis–DNA structure with residues involved in DNA contact depicted in stick format. Residues are colored as in (**A)**, with N84 (orange) being replaced by serine in the NinH sequence. The orientation of the complex differs slightly from (**C)** to make it easier to visualize the contacts with DNA. Key guanosine and cytosine base pairs recognized by Fis are indicated at −7 and 7.

A search for previously uncharacterized λ genes likely to participate in phage DNA metabolism uncovered the 68-residue *ninH* gene product as a likely candidate due to the presence of a putative HTH motif. Residues 17–38 in NinH were predicted to form a HTH using two different search methodologies [38,39]. NinH belongs to the Protein Family PF06322 [40] as a domain of unknown function incorporating 346 entries. Members of the family are exclusively linked to phage and prophage sequences, with the former found primarily among the Siphoviridae and Podoviridae. Position-specific iterative BLAST analysis [41], alongside Phyre^2^ [42] and I-TASSER [43] structural homology searches with NinH, uncovered significant matches with three helices spanning the HTH motif in the *E. coli* Fis protein (Figure 1A and Supplementary Figure S2) and other transcriptional regulators, including homologs from the NtrC and LysR families. The ancestral relationship between Fis and NtrC has been noted previously [44]. Using Phyre^2^, Fis yielded the highest match with 91.9% confidence over 32 amino acids at the N-terminus of NinH (residues 6–37). This region overlaps with the HTH helix motif in Fis that spans helices C and D and corresponds to the DNA binding site [14]. *E. coli* Fis shares 40% identity and 67% similarity with NinH over 30 residues in this region (Figure 1A). Strikingly, seven out of eight residues that are known to contact DNA from the crystal structure of the bipartite complex [14] are conserved in λ NinH (Figure 1A–D). The single exception is Asn84 in *E. coli* Fis which corresponds to Ser29 in NinH; although this residue is dispensable for recognition of high-affinity sites [45]. NinH lacks the N-terminal section present in Fis and has an extended C-terminus (Figure 1A). Residues 44–48 and 51–55 are predicted by JPred4 [46] and other secondary structure prediction tools to form β sheets and these could be analogous to those found at the N-terminus of Fis (Figure 1C). Overall, the bioinformatic analysis is consistent with NinH being a short, DNA binding protein related to *E. coli* Fis.

### NinH structure

While the bioinformatic analysis outlined above supports structural homology between the C-terminal DNA binding region of Fis and the N-terminal portion of NinH, questions remain over the structure and role of the NinH C-terminus and its potential involvement in dimerization. To address this, we attempted to produce an *in silico* structural model of a NinH dimer which was further validated using SAXS.

**Figure 2.**
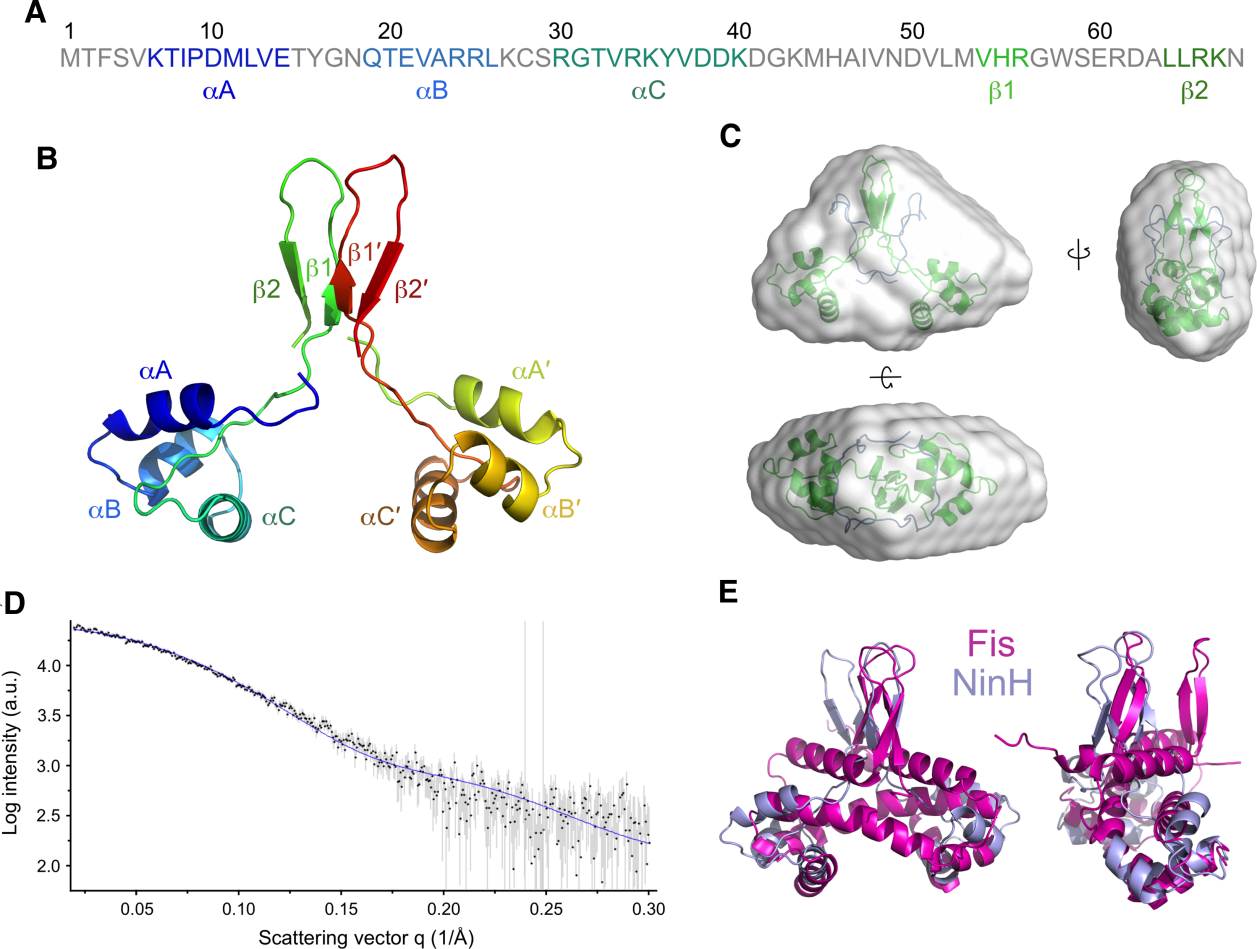
Modeling and structural analyses of NinH. (**A**) Primary sequence of λ NinH protein highlighting the predicted secondary structure elements. (**B**) Modeled structure of the NinH dimer with secondary structure features indicated. (**C**) Top, front and side views of the NinH dimer model (green) with N-terminal His-tags (blue) fitted into the low molecular mass envelope generated from the SAXS profile. The high-resolution model fits the triangular prism shape well with some empty space suggesting flexibility of the N-terminal His-tags. (**D**) Overlay of SAXS scattering profile of His-NinH (black dots) and putative scattering profile generated from the high-resolution model using Crysol software (blue). The overall quality of the fit is high (χ^2^- test value of 1.03). (**E**) Front and side views of the structural alignment of the NinH modeled complex with Fis (PDB: 3IV5).

For the *in silico* model, based on biochemical evidence presented below that the C-terminal region contributes to dimer formation, we chose a model that assumed the involvement of His55 and Arg56 and used these residues as a constraint in HADDOCK. The resulting model (Figure 2A,B) had a ‘closed' conformation and an RMSD of ∼5.6 Å when compared with the Fis structure and ∼4.8 Å when only the residues of DNA binding region and the chosen model are considered. From the library of potential structures obtained, we noted that nearly all gave a similar structure to the αD helix in Fis corresponding to the DNA binding region (NinH residues 29–35). The C-terminal regions of the NinH protein were consistently predicted to form a β sheet structure, as found in the N-terminal region of Fis. In Fis, these regions are adjacent to the dimer interface formed by the interlocking of helices A and B [14], i.e. are in a ‘closed' conformation. Our modeling generated a range of conformations from wide open to closed states (6.7 Å between C-terminal loop tips for closed states and 11.2 Å for open; Supplementary Figure S3A). It should be noted that NinH residues 44–48 predicted to form a β sheet (Supplementary Figure S2A) are not part of the hairpin structure in the modeled dimer.

To see if the model fitted experimentally obtained data, we carried out a structural analysis of purified His-NinH using SAXS. SAXS is a powerful structure determination method that does not require labeling or crystal formation, but the resulting structural information is of low resolution. It is, however, an excellent validation tool for high resolution *in silico* modeling. The structural properties resulting from SAXS analysis confirmed NinH to be a globular dimer with a molecular mass of ∼20 kDa, as expected (Supplementary Figure S3B). To generate such a model, we firstly built the NinH dimer *de novo* as described in the methods section. The chosen model fits well into the low molecular envelope that was generated solely from SAXS data (Figure 2C), further validating the generated model. It also fits well in the Pearson's χ^2^-test between the putative scattering curve generated from this model and SAXS experimental data as calculated from Crysol software [47]; Figure 2D and Supplementary Figure S3B,D). Moreover, the alignment of this monomer on the structure of the Fis dimer (Figure 2E) shows a good overall fit suggesting that NinH resembles Fis in quaternary structure.

### NinH dimer formation

Fis is a dimer in solution allowing the HTH domain from each subunit to dock within the major groove of duplex DNA [14]. Both native and N-His-tagged NinH proteins were purified to homogeneity (Supplementary Figure S4A,B). The histidine tag could be readily removed using thrombin protease (Supplementary Figure S4B). Stable protein dimers can survive separation in SDS-polyacrylamide gels if reducing agents are omitted and the sample is not boiled [48]. His-NinH was analyzed using this approach and a dimer band of ∼20 kDa was evident in Coomassie-stained gels (Supplementary Figure S4B), which was confirmed by Western blotting using anti-His antibodies (Supplementary Figure S4C). Dynamic light scattering was also performed with His-NinH, which showed an increase in particle size with increasing protein concentration, also consistent with dimerization (Supplementary Figure S4D). Finally, His-NinH was subjected to AUC to further help determine its quaternary structure. The His-NinH protein sedimented in a single peak at 20.2 kDa (Supplementary Figure S4E), matching the predicted molecular mass of a homodimer (20 kDa).

### NinH DNA binding

**Figure 3.**
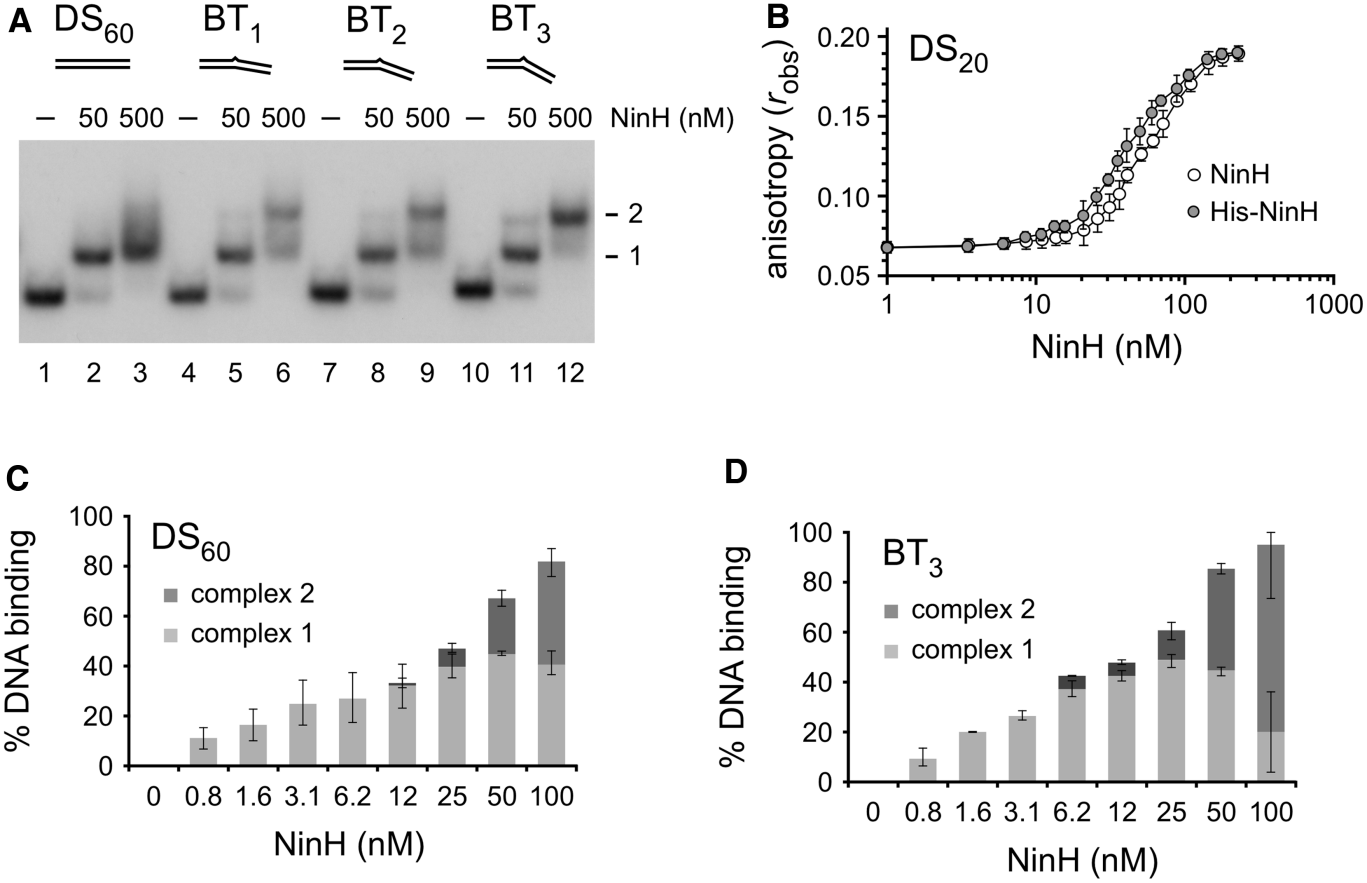
DNA binding by NinH protein. (**A**) Gel mobility shift assays contained 50 or 500 nM NinH protein and 0.15 nM of ^32^P-labeled 60 bp (DS_60_) dsDNA (lanes 1–3) and bent DNA (BT_1–3_) containing 1 (lanes 4–6), 2 (lanes 7–9) or 3 (lanes 10–12) additional adenine residues in one of the DNA strands. (**B**) NinH and His-NinH binding to 10 nM fluorescein-labeled 20 bp dsDNA (DS_20_) as determined by fluorescence anisotropy. Data are the mean and standard deviation of two (His-NinH) and three (NinH) independent experiments. (**C**,**D**) NinH binding to linear dsDNA and bent DNA. Binding reactions contained 0.15 nM DNA with 0.781, 1.562, 3.125, 6.25, 12.5, 25, 50 and 100 nM protein. Data are the mean and standard deviation of three independent experiments. The relative proportion of the two protein–DNA complexes was determined by ImageJ [68] analysis of phosphorimaged gels.

The predicted interaction of NinH with DNA was initially evaluated in an electrophoretic mobility shift assay with short 5′-^32^P-radiolabeled substrates (Figure 3A). Purified NinH bound to a 60 bp dsDNA (DS_60_; Supplementary Table S1) forming two complexes (Figure 3A, lanes 1–3; Supplementary Figure S5), confirming that it is capable of forming a stable association with DNA. To quantify this binding affinity of NinH for dsDNA we utilized fluorescence anisotropy of a 3′-fluorescein-tagged 20 bp duplex (DS_20_; Figure 3B), which yielded an estimated *K*_D_ of 57 nM for a NinH dimer. Interestingly, the best fit for the data is produced with a Hill slope coefficient of 2.23, implying cooperativity of binding to DNA. Similar results were obtained with His-NinH binding to DS_20_ in yielding a *K*_D_ of 43 nM and a Hill slope coefficient of 1.985. DNA substrates that resemble branched molecules that might occur as intermediates during λ DNA metabolism, a replication fork-like structure and a four-stranded Holliday junction were also tested, but no significantly improved His-NinH binding was detected with complexes appearing simply to assemble on available duplexes (Supplementary Figure S5A). Similarly, no enhanced affinity was observed with bubble DNA substrates containing centrally located mismatches of 1, 5, 13 and 20 nt (Supplementary Figure S5B).

Since Fis is known to bend duplex DNA [14,16,17], we explored the possibility that NinH might bind with a higher affinity to a duplex that already contained a kink. Three bent dsDNA substrates (BT_1–3_) were generated by the addition of extra adenine residues at the center of one strand of the duplex (Supplementary Table S1), causing it to kink to accommodate the extra nucleotide. Adenine insertions of 3 nt are known to introduce a bend of ∼60° in duplex DNA [49]. NinH bound to all three of the bent DNA structures tested, forming two discrete complexes (Figure 3A, lanes 4–12). There was little difference in apparent binding affinity between DS_60_ and BT_1–3_, although the bent DNA did allow more of the second complex to form (Figure 3C,D). The complexes observed in these gel shift assays most probably represent the initial assembly of a single NinH dimer followed by the cooperative loading of a second dimer, a feature apparently favored when the duplex already contains a bend.

### NinH DNA bending

**Figure 4.**
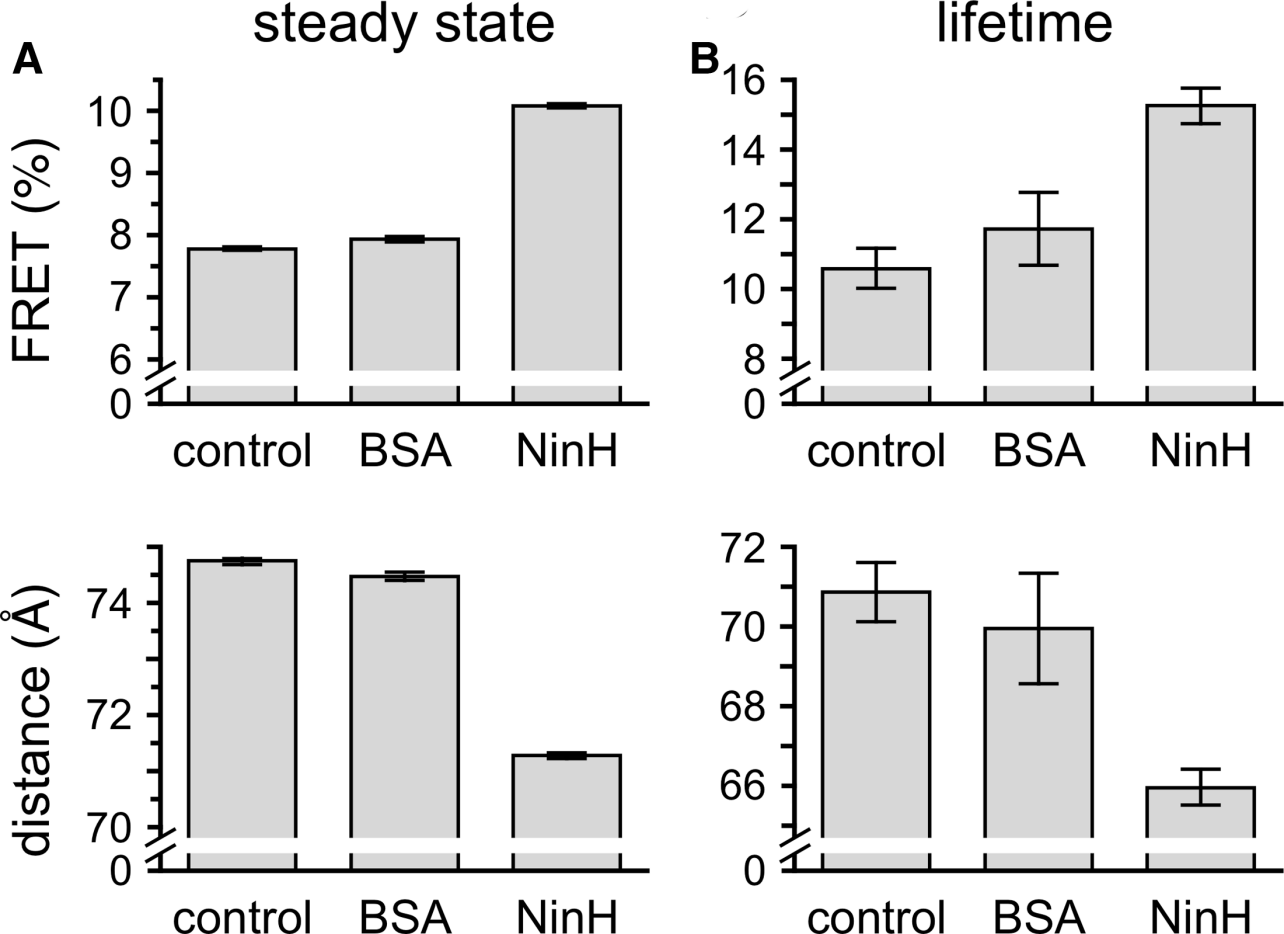
Bending of dsDNA by NinH. (**A**,**B**) Energy transfer was determined in steady state and lifetime FRET assays. Reactions contained 1 μM NinH protein or controls without protein or addition of 3 µM BSA with 50 nM DNA and were incubated for 10 min at room temperature before analysis. Experiments were performed in triplicate; error bars indicate the standard error of the mean. Negative controls have been used previously [69].

In keeping with its similarities to Fis, we next examined the capacity of NinH to induce a bend in linear duplex DNA. A 20 bp substrate (DS_20_), matching that employed in fluorescence anisotropy binding assays, was generated by annealing oligonucleotides labeled with either tetramethylrhodamine or fluorescein at the 3′ end. ssFRET was determined from the increase in sensitized acceptor emission. The energy transfer efficiency increases upon the addition of NinH (Figure 4A) consistent with a shortening of the inter-fluorophore distance. No significant difference in FRET was observed between controls without protein or with BSA. The divergence between NinH and the controls is particularly evident in the raw data for fluorescence intensity (Supplementary Figure S6). Energy transfer was independently assayed by time-resolved fluorescence spectroscopy. As with the steady-state assay, energy transfer efficiency also increased upon the addition of NinH, with a more substantial increase noted (Figure 4B) compared with the BSA and protein-free controls. From the energy transfer efficiencies, donor to acceptor distances were calculated (Supplementary Table S2). These data clearly show a decrease in donor to acceptor distance in the presence of NinH. Accordingly, the mean percentage change in energy transfer distance was calculated as −4.63 for the steady-state assay and −8.03 for the time-resolved. The discrepancy in the absolute values of R calculated for the two methodologies has been noted previously and many explanations have been proposed [50,51]. Both steady-state and time-resolved data were employed in calculations to estimate the extent of bending induced by NinH binding to duplex DNA (Supplementary Table S3).

The calculations based on the two methods yielded values of 46° and 35° for steady-state and time-resolved FRET, respectively (Supplementary Table S3). Averaging these values gives a bend of 41° which is somewhat lower than the 50–90° estimated for Fis in gel and footprinting experiments [16,52] or 65° from the crystal structure of Fis bound to a high-affinity DNA site [14]. Thus NinH resembles Fis in being capable of bending dsDNA, although the reduced bend with NinH suggests a difference in the arrangement of the dimer in its association with duplex DNA.

### NinH sequence specificity

**Figure 5.**
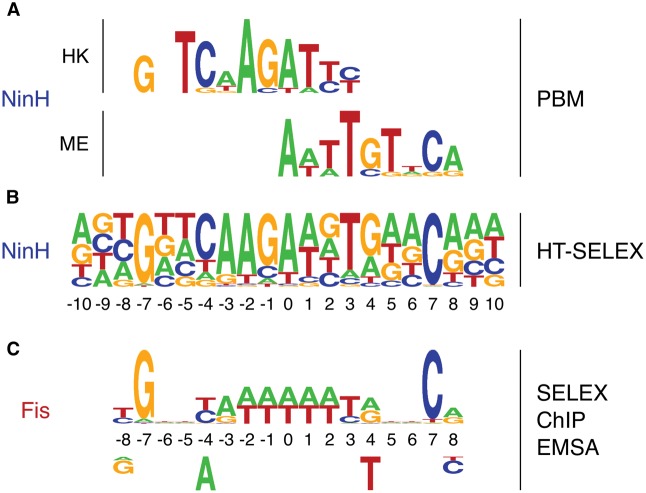
Identification of the NinH DNA binding motif. (**A**) The GST-NinH binding site obtained by PBM using HK and ME microarrays. (**B**) The GST-NinH binding motif determined by HT-SELEX. (**C**) Published Fis DNA binding site based on SELEX, ChIP and EMSA data [3,13–15]. Residues below the motif correspond to those that exert a negative effect on Fis binding. Motif residues in (**A**) and (**B**) are numbered and aligned according to the Fis recognition sequence shown in (**C**) and are also highlighted in Figure 1D. Motif alignments were performed manually.

To determine whether NinH displays any preference for particular nucleotide sequences we first conducted universal PBM assays, which comprehensively survey 10-base sequences and provide robust estimates for binding to all possible 8-mers, since each is measured in multiple contexts [30]. An N-terminal GST-NinH fusion was constructed under the control of the vector T7 promoter and protein produced *in vitro*. Preferential sequence binding with the fusion protein was performed in duplicate on two microarrays with differing dsDNA probe sequences (denoted ME and HK; [31]). The results on both arrays were consistent with established metrics for sequence-specific binding ([30]; Supplementary Figure S7), but the highest-scoring sequences were dissimilar between the two arrays. A position-specific scoring matrix (PSSM) sequence logo of the top ten 8-mers recovered from each array is shown in Figure 5A.

The differing results from the two arrays suggest that each array samples from a larger binding site: while the arrays comprehensively sample 10-base sequences, their sampling of larger sequences (typical of bacterial transcription factors [53]) is much sparser and may not capture full binding sites; the Fis footprint, for example, is 19–21 base pairs [14]. High-throughput selection (HT-SELEX) was, therefore, used in an effort to more clearly define the sequence composition of any NinH DNA binding motif. The results revealed a degenerate motif with a clear 5′-G-N_13_-C-3′ signature (Figure 5B). The palindromic nature of the motif fits with the assembly of a NinH homodimer with each HTH domain engaging with the major groove. Moreover, the larger motif detected by HT-SELEX can also explain the PBM data, revealing that the ME and HK motifs correspond to the left and right ends of the NinH recognition sequence (Figure 5A). Remarkably, in several aspects, the motif closely resembles the Fis DNA binding site (Figure 5C) identified by a combination of SELEX, CHiP and gel shift experiments [3,13-15]. Both NinH and Fis binding sites have G and C separated by 13 nt, plus additional matches at −8/8, −4/4 and −3/3 (Figure 5B,C). Thus the homology model of NinH based on Fis and supported by SAXS data (Figure 2) fits with a similar arrangement in the two proteins with specific contacts with guanines at each end of the motif (Figure 1D). However, there are differences in the motifs between Fis and NinH in the core 5 bp from −2 to 2 (Figure 5B,C). In the Fis motif, this region is AT-rich [13,14], whereas NinH shows a distinctly different signature, notably with a G or C at the −1 position and no preference for an A or T at position 2 (Figure 5B). Given the differences in the central core recognition sequences, it is possible that NinH and Fis do not assemble preferentially at precisely the same sites although it is likely that there will be overlap in those they favor.

### NinH site-directed mutants

**Figure 6.**
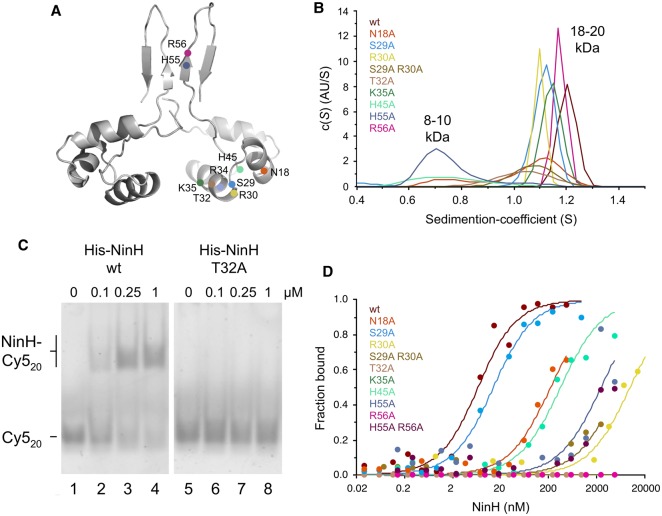
Oligomeric state and DNA binding properties of NinH site-directed mutants. (**A**) Structure of the modeled NinH dimer highlighting the position of residues mutated to alanine. (**B**) Analytical centrifugation analysis assessing the monomeric or dimeric state of NinH proteins in solution. Peaks are labeled with the calculated molecular mass. (**C**) DNA binding by His-NinH wt and T32A proteins. Gel shift assays were performed with 10 nM Cy5-labeled 20 bp dsDNA (Cy5_20_) matching the NinH consensus sequence (Supplementary Table S1). DNA–protein interactions were assessed by 7% neutral PAGE. (**D**) NinH mutant binding assessed by thermophoresis using 10 nM Cy5_20_ dsDNA.

To further probe the similarities between NinH and Fis we selected many residues for replacement by alanine to investigate regions of NinH implicated in DNA binding and dimerization (Figure 6A). We initially selected conserved residues corresponding to the HTH of Fis, namely N18, R30, T32, R34 and K35 matching Fis N73, R85, T87 R89 and K90, respectively (Figures 1 and 6A). S29 was also chosen as it corresponds to Fis N84, the only residue involved in DNA contact that is not conserved between Fis and NinH (Figure 1). Additional substitution mutants (H45A, H55A and R56A) were generated in the predicted β sheets at the C-terminus of NinH proposed to be involved in dimerization. A C-terminal deletion (ΔC25, removing residues 44–68) and two double mutants (S29A R30A and H55A R56A) were also constructed (Supplementary Figure S1). All of the N-His-tagged NinH proteins were purified to homogeneity, with the exception of R34A and ΔC25 which could not be retrieved due to insolubility.

#### DNA binding mutants

N-terminal mutants affecting the predicted DNA binding region of NinH were initially checked for their ability to form homodimers in solution using AUC (Figure 6B). All of these mutants form dimers, although N18A shows an additional peak at 8–10 kDa consistent with the formation of a small proportion of monomer. We proceeded to investigate whether any of these mutants were impaired in DNA binding. A gel shift assay with a Cy5-labeled 20 bp dsDNA sequence (Cy5_20_) that matches the NinH consensus sequence derived from HT-SELEX experiments (Figure 5B) was employed. The His-NinH wt protein bound this DNA with a *K*_D_ in the sub-micromolar range (Figure 6C, lanes 1–4). This binding affinity was further quantified using MST, which yielded a *K*_D_ of 5.6 nM (Table 1 and Supplementary Figure S8). Both gel shift and MST experiments were repeated with the NinH site-directed mutants and key residues involved in DNA binding identified (Table 1, Figure 6D and Supplementary Figure S9). The most important residue for DNA binding appears to be T32 (Table 1, Figure 6C and Supplementary Figure S8C,D), equivalent to T87 in Fis (Figure 1D) where it is known to contact a phosphate on the DNA backbone and is similarly critical for DNA binding [14,45]. The T32A mutant also exhibited a defect in the bending of the DS_20_ substrate (Supplementary Figure S10). Other NinH mutants with significant deficiencies in DNA binding were R30A (Fis R85), K35A (Fis K90) and the double mutant S29A R30A, all increasing the *K*_D_ by at least three orders of magnitude (Table 1). The N18A (Fis N73) had an intermediate effect on DNA binding (Figure 6D). Mutation of S29 (Fis N84) to alanine had only a minor effect on DNA binding (Table 1). A Fis N84A mutant shows reduced binding to λ *att*R, but is largely unaffected in binding to *fis* P II and *hin* distal sites [45]. All of these NinH mutants exhibited DNA deficiencies that broadly match the effects conferred by their corresponding mutants in the αC and αD helices of the Fis HTH (Table 1; [45]).

**Table 1.** DNA binding of His-NinH mutants

NinH mutant	*K*_D_ on Cy5_20_	Fis mutant	*K*_D_ on λ *attR*
wt	5.6 ± 1.5	wt	4.1 ± 0.4
N18A	>220	N73A	91 ± 34
S29A	13 ± 2.7	N84A	157 ± 15
S29A R30A	>5500	—	—
R30A	>9900	R85A	>2200
T32A	*No binding*	T87A	1300** **± 545
K35A	*No binding*	K90A	>2200
H45A	>350		
H55A	>2500		
R56A	*No binding*		
H55A R56A	*No binding*		

Dissociation constants (*K*_D_) in nM were determined by microscale thermophoresis and are the mean and standard deviation of three independent experiments. Binding mixtures contained 10 nM Cy5_20_ DNA. No DNA binding was detected for mutants T32A, K35A and R56A under these conditions. Similarly, only weak binding was found with H55A R56A and the binding isotherm could not be used to estimate a *K*_D_. The λ *att*R binding data on Fis mutants equivalent to residues mutated in NinH are taken from Feldman et al. [45].

#### Mutants affecting dimerization

Three substitution mutants, H45A, H55A and R56A, predicted to be involved in dimerization were also purified and analyzed by AUC (Figure 6B). H45A and H55A produced a single peak at 8–10 kDa consistent with the mass of a NinH monomer. These results are supportive of the structural model proposed for NinH whereby the C-terminus contributes to intersubunit interactions. In contrast, the R56A mutant located at the distal end of β1/β1′ (Figure 2B) was able to form a stable dimer (Figure 6B), but was unable to associate with DNA (Figure 6D). It may be that the R56A mutation affects the optimal positioning of the two HTH domains. H55A also bound poorly to DNA, but H45A, which is a monomer in solution, retained significant binding capability (Table 1; Figure 6D; Supplementary Figure S9). It may be that the presence of DNA brings individual monomers in close enough proximity to promote dimerization. The H55A R56A double mutant displayed very poor DNA binding as with the two individually mutated NinH proteins (Figure 6D).

### NinH toxicity

**Figure 7.**
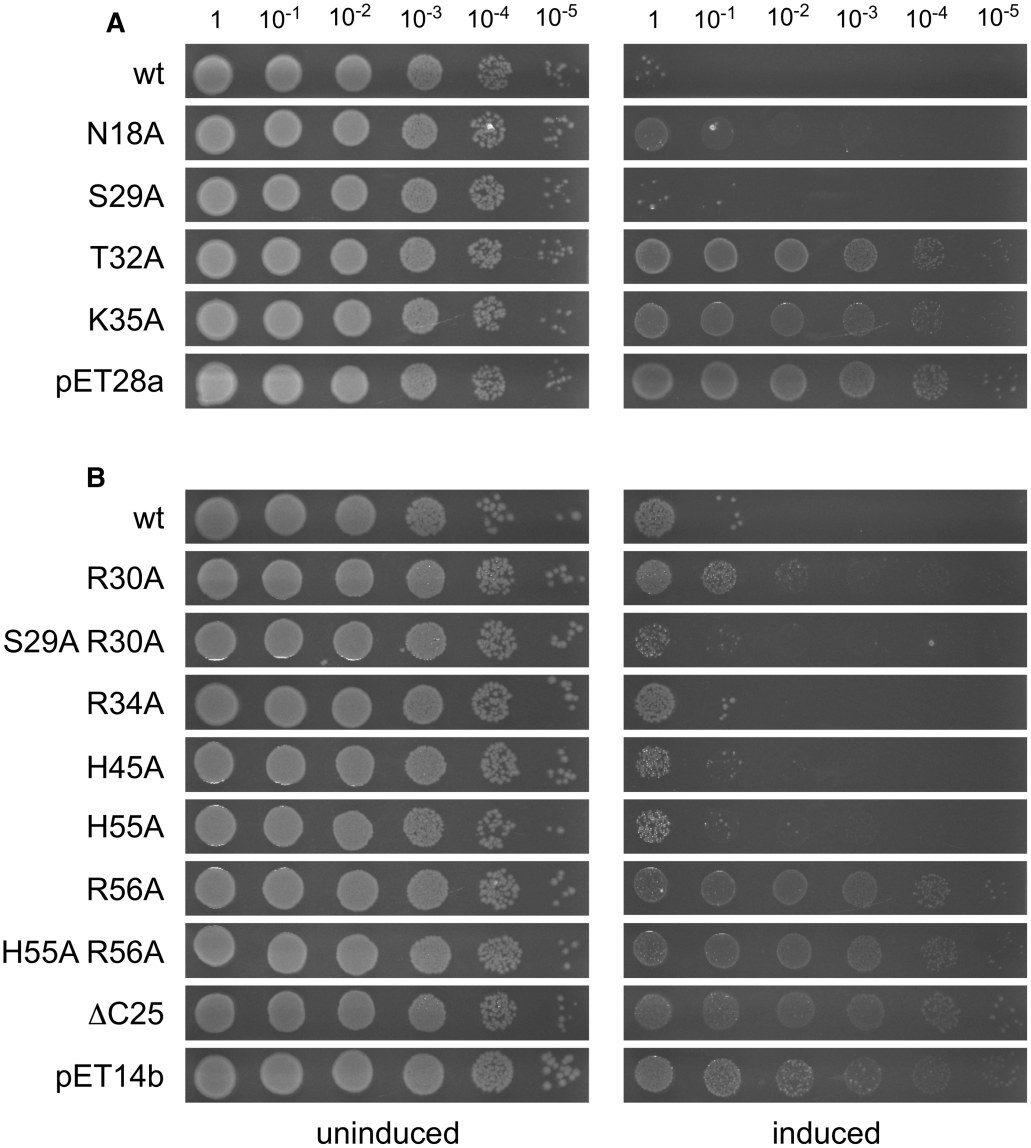
Effect of NinH wt and mutant overexpression on *E. coli* growth. BL21-AI carrying pHis-NinH, pHis-NinH mutants and appropriate vector controls, pET28a(+) and pET14b, were grown to an A_650nm_ of 0.4 in LB at 37°C. Serial dilutions (10 µl) were spotted onto agar plates containing either (**A**) kanamycin or (**B**) ampicillin without (uninduced) or with the addition of 0.2% arabinose and 1 mM IPTG (induced).

We next examined the effect of His-NinH wt and mutants on *E. coli* BL21-AI by plating dilutions of exponentially growing cells on media containing both arabinose and IPTG. Under conditions which induced overexpression, both His-NinH wt constructs severely inhibited bacterial growth when compared with either the pET28a or pET14b vector controls (Figure 7). In the absence of the two inducer molecules, cells carrying each of the plasmids yielded a normal growth phenotype (Figure 7). We considered it likely that NinH binding to bacterial DNA was responsible for toxicity by interference with normal chromosome duplication and segregation. If this is correct, then bacterial growth inhibition could be used to monitor DNA binding of the NinH mutants *in vivo*. High-level production of all of the mutant NinH proteins was confirmed following the activation of gene expression (Supplementary Figure S11). In these toxicity assays, we found that mutants defective in DNA binding exerted little or no detrimental growth effect (T32A and K35A) whereas those that retained DNA binding, such as S29A, behaved like the wt when induced (Figure 7A). Similarly, N18A which has reduced DNA binding activity displayed an intermediate growth defect (Figure 7A). R34A, which could not be purified, resembled the wt suggesting that this protein retains some DNA binding activity, which would be in keeping with the equivalent Fis mutant, R89A [45]. R30A allowed improved growth but when combined with S29A exhibited a more severe growth effect, which is surprising given that R30A and the double mutant, S29A R30A, both bind DNA poorly. There may be a difference in specific versus non-specific binding in some of these mutants that confer differing *in vivo* effects [45]. Finally, dimerization mutants that were unable to bind DNA, R56A and H55A R56A, did not cause a growth defect (Figure 7B). H45A, which exhibits an intermediate level of DNA binding was toxic. H55A does not bind DNA well and did show some improved growth, though it was still relatively weak. The mutant lacking the C-terminus (ΔC25) showed no toxicity consistent with an expected inability to dimerize or associate with DNA (Figure 7B). Overall, the toxicity assays align well with the DNA binding results, linking affinity with DNA substrates *in vitro* and chromosomal DNA *in vivo*.

## Discussion

The function of the previously uncharacterized phage λ NinH protein was investigated and our results reveal that it resembles bacterial Fis in predicted structure, ability to form homodimers and in bending double-stranded DNA. The conservation of primary sequence between NinH and Fis DNA binding domains suggests that both bind and bend DNA in an analogous fashion. Non-specific DNA binding was apparent with NinH, a feature shared by Fis and other NAPs [14,15], while no increased affinity for bubble, fork or Holliday junction DNA structures was detected. Dual assembly of NinH on duplexes that already contain a kink was favored, potentially overcoming an energetic barrier to bending; it is possible that these substrates more closely resemble the end product of conventional binding by the phage protein. Intrinsic DNA bending does not appear to contribute significantly to the recognition of preferred sites by Fis [14], though flexibility in residues flanking the 15 bp consensus is important [15]. Other NAPs, notably IHF, HU and H-NS, are known to preferentially associate with curved, bent and distorted DNA structures [54,55]. Using a FRET assay, the extent of DNA bending induced by NinH on a non-cognate site was found to be ∼41°, 24° less than that imposed by Fis on its preferred consensus sequence [14].

NinH shared a preference with *E. coli* Fis for a pseudo-palindromic consensus binding site containing G and C residues flanking a 13 bp core. Although within the central region their motifs differ slightly, there is a clear pyrimidine–purine pairing at positions −4/4 and −3/3 (Figure 5) that is similar to that also seen with Fis and helps binding by its enhanced propensity for bending [14]. Fis prefers sites that have an AT-rich core, with the highest affinity for a central A_4–6_ tract [3,14]. In contrast, the NinH motif has G/C at −1 and no preference for any residue at position 2 (Figure 5). This variation in the central motifs probably reflects preferences for sequences that affect localized DNA bending and minor groove width, known to influence Fis binding affinity [15]. It is possible that NinH makes base-specific contact with residues in this region, although this would mean that the phage protein has a radically different configuration relative to Fis. The differences in Fis and NinH motifs may not be significant and both proteins could potentially compete for the same nucleotide sequence targets [14,15]; Asn84 in Fis (Ser29 in NinH) makes base-specific contacts at −4/4 but the consensus sequences match at this position (Figure 5B,C); neither this residue nor other internal nucleotide sequences are critical for Fis binding at high-affinity sites [14,45]. In keeping with this, a NinH S29A mutant displayed only a modest reduction in DNA binding to a sequence matching the NinH consensus. Given that NinH and Fis must differ in the way that they assemble as homodimers, it is remarkable that the signature sequences they recognize are not more divergent.

Sequence alignments and structural predictions combined with DNA binding analyses to support a model whereby the N-terminal domain of NinH consists of three α helices, analogous to αB, αC and αD in Fis that comprise the HTH domain (Figure 1). The structural model of the predicted NinH DNA binding region was confirmed experimentally using site-directed mutants which confirmed the importance of conserved residues equivalent to those known to be responsible for DNA binding by Fis [14,45]. These results also correlated with *in vivo* toxicity, with mutants defective in DNA binding lacking the growth inhibition phenotype associated with NinH overexpression. The ensemble *in silico* models we generated show a similarly conserved secondary structure. The tertiary structure of α helical regions is also retained but the positioning of the C-terminal regions on one monomer relative to the DNA binding regions and relative to the same regions of the partner monomer were found to vary model-to-model. While we chose a ‘closed' conformation of the C-terminal regions consistent with it forming a dimer interface and found that this fits the SAXS data well, alternative conformations cannot be ruled out. Furthermore, higher resolution data will be required to place the C-terminal regions unambiguously. Biophysical studies confirmed that NinH is a homodimer in solution and that residues outside of the DNA binding region appear to contribute directly or indirectly to dimerization. The structure of the C-terminal region of NinH implicated in dimer formation has proven more challenging to predict, particularly in its spatial relationship with the N-terminal region. All predictive models suggest that this region contains β sheets. Mutation of conserved residues predicted to lie in these β sheets (residues 45 and 55) resulted in a shift in the monomer-dimer equilibrium towards monomer consistent with a role in the dimer interface as suggested by its position in our favored model. A similar shift is seen for the protein with a mutation at residue 45 but on our model this does not appear to be at the dimer interface, implying an indirect effect.

The remarkable similarities in NinH and Fis DNA binding and bending, mean that they have the potential to be functionally equivalent factors affecting several aspects of λ and *E. coli* biology. Fis fulfills an accessory role in excision of the λ prophage by manipulating *att*L and *att*R into an appropriate position for cleavage by integrase (Int) in complex with Xis bound to *att*R [6,7,11,56]. Fis binding at this site also contributes to phage integration [9–11]. NinH is expressed early, along with the other *ninR* genes exclusively during the λ lytic cycle under *P*_R_ transcriptional control, with trace levels appearing 2 min after prophage induction and high-level expression from 5 to 20 min [57]. This pattern of expression mirrors that of *int* and *xis* which are transcribed from *P*_L_ [57], meaning that NinH could participate in the excision process in addition to integration. It is plausible that NinH and Fis can compete for binding at *att*R to influence the efficiency of these site-specific recombination reactions. It may, therefore, be significant that the Fis binding site in *att*R contains an A_6_-tract (position -3 to 2) rather than the 5′-AAGAAN-3′ signature found with NinH (Figure 5). Following phage induction, Fis might be expected to predominate as it is abundant in *E. coli* during early exponential growth and is already available in the cell. However, the physiological condition of the cell might be a contributing factor since Fis expression is barely detectable during the stationary phase [58]. It is also feasible that NinH exerts influence on bacterial cells by interfering with the expression of genes, especially since Fis is a major factor in global transcriptional regulation, affecting the expression of 923 genes [3]. NinH could compete with Fis to affect specific regulatory pathways, or even nucleoid architecture, with specific advantages for phage productivity. Interestingly, a precise *ninH* gene deletion results in a phage that forms small plaques when grown on *E. coli* [59], implying an important role in maximizing phage yield or efficiency of lysis.

Several examples of NAP-like functions have previously been found associated with extrachromosomal elements [60], including *Bacillus subtilis* phage ϕ29 p6 [61] and homologs of H-NS present in phage EPV1 [62] and conjugative plasmids R446 [63] and pSf-R27 [64]. H-NS is primarily a repressor of gene expression [65] and selfish DNA elements carrying versions of the protein may be harnessed to down-regulate bacterial defense genes to promote their persistence [64]. For example, the plasmid-encoded Sfh protein resembles H-NS and StpA [66]. The occurrence of phage-encoded anti-repressors of H-NS [67] also supports the notion that phages undertake a variety of approaches to modulate host NAPs and subvert host regulatory pathways to promote their own survival. NinH is the first example to be identified of a Fis-like NAP carried by bacteriophages. Further work is needed to determine the precise role of NinH in both phage and bacterial metabolism.

## Abbreviations

AUC: analytical ultracentrifugation
FWHM: full width half maximum
HTH: helix-turn-helix
IRF: instrument response function
NAPs: nucleoid-associated proteins
PBM: protein binding microarray
SAXS: small angle X-ray scattering
ssFRET: steady-state Förster resonance energy transfer
trFRET: time-resolved Förster resonance energy transfer
