# Supplementary material for: A bacteriophage mimic of the bacterial nucleoid-associated protein Fis
Source: Biochem J. 2020 Apr 17;477(7):1345–62. doi: 10.1042/BCJ20200146 (PMC7166090; doi:10.1042/BCJ20200146)
Supplement: Supplementary Tables S1-S3 and Figures S1-S11 [file BCJ-477-1345-s1.pdf]

Supplementary Information

A bacteriophage mimic of the bacterial nucleoid-associated protein Fis

Soumyananda Chakraborti, Dhanasekaran Balakrishnan, Alexander J. Trotter, William H. Gittens, Ally W.H. Yang, Joy R. Paterson, Sylwia Świątek, Jacek Plewka, Fiona, A. Curtis, Laura Y. Bowers, Lars-Olof Pålsson, Timothy R. Hughes, Michał Taube, Maciej Kozak, Jonathan G. Heddle and Gary J. Sharples

Table S1. Oligonucleotides used to generate DNA substrates.

Oligo	Nucleotide sequence (5′-3′)
o-SS$_{60}$	TTTGGTCTAACTTTACCGCTACTAAATGCCGCGGATTGGTTTCGCTGAATCAGGTTATTA
o-DS$_{60}$	TAATAACCTGATTCAGCGAAACCAATCCGCGGCATTTAGTAGCGGTAAAGTTAGACCAAA
o-BT$_1$	TAATAACCTGATTCAGCGAAACCAATCCAGCGGCATTTAGTAGCGGTAAAGTTAGACCAAA
o-BT$_2$	TAATAACCTGATTCAGCGAAACCAATCCAAGCGGCATTTAGTAGCGGTAAAGTTAGACCAAA
o-BT$_3$	TAATAACCTGATTCAGCGAAACCAATCCAAAGCGGCATTTAGTAGCGGTAAAGTTAGACCAAA
o-BB$_1$	TAATAACCTGATTCAGCGAAACCAATCGGCGGCATTTAGTAGCGGTAAAGTTAGACCAAA
o-BB$_5$	TAATAACCTGATTCAGCGAAACCAATCGCAACCATTTAGTAGCGGTAAAGTTAGACCAAA
o-BB$_{13}$	TAATAACCTGATTCAGCGAAACATTGTAGGTAAGCTTAGTAGCGGTAAAGTTAGACCAAA
o-BB$_{20}$	TAATAACCTGATTCAGCGAATGACCGATAACGTCCACTTGAGCGGTAAAGTTAGACCAAA
o-SS$_{20}$	TCTTTGCCCACGTTGACCCA
o-FLU$_{20}$	TGGGTCAACGTGGGCAAAGA-fluorescein
o-TET$_{20}$	TCTTTGCCCACGTTGACCCA-tetramethylrhodamine
o-HJ-1$_{50}$	GGCGACGTGATCACCAGATGATTGCTAGGCATGCTTTCCGCAAGAGAAGC
o-HJ-2$_{50}$	GGCTTCTCTTGCGGAAAGCATGCCTAGCAATCCTGTCAGCTGCATGGAAC
o-HJ-3$_{50}$	GGTTCCATGCAGCTGACAGGATTGCTAGGCTCAAGGCGAACTGCTAACGG
o-HJ-4$_{50}$	ACCGTTAGCAGTTCGCCTTGAGCCTAGCAATCATCTGGTGATCACGTCGC
o-DS$_{50}$	GCTTCTCTTGCGGAAAGCATGCCTAGCAATCATCTGGTGATCACGTCGCC
o-Cy5$_{20}$	cyanine5-ACCGATCAAGAATTGTGCAT
o-cCy5$_{20}$	ATGCACAATTCTTGATCGGT

The 60 nt ssDNA substrate (SS$_{60}$) consisted of o-SS$_{60}$, which was annealed to its complement (o-DS$_{60}$) to give a fully complementary 60 bp dsDNA duplex (DS$_{60}$). Bent DNA substrates containing 1 (BT$_1$), 2 (BT$_2$) or 3 (BT$_3$) adenine insertions were made by annealing o-SS60 with o-BT$_1$, o-BT$_2$ and o-BT$_3$, respectively. Bubble (BB) structures of 1, 5, 13 and 20 nt (BB$_1$, BB$_5$, BB$_{13}$ and BB$_{20}$) were made by annealing o-SS$_{60}$ with o-BB$_1$, o-BB$_5$, o-BB$_{13}$ and o-BB$_{20}$, respectively. o-HJ-1$_{50}$ was annealed to o-DS$_{50}$ to give a 50 bp dsDNA duplex, to o-HJ-2$_{50}$ to give a fork structure and to o-HJ-2$_{50}$, o-HJ-3$_{50}$, and o-HJ-4$_{50}$ to give a Holliday junction with an 11 bp homologous core. Nucleotides underlined in BT and BB oligonucleotides indicate sequences that generate insertions or bubble structures, respectively, when annealed to o-SS$_{60}$. Those underlined in the HJ oligonucleotides indicate the position of the 11 bp homologous core of the Holliday junction structure. o-Cy5$_{20}$ when annealed to its complement (o-cCy5$_{20}$) provided a duplex that matches the NinH preferred target consensus sequence. In gel shift assays, oligonucleotides o-SS$_{60}$ or o-HJ-1$_{50}$ were labeled with [γ^{32}P] ATP (Perkin-Elmer) at the 5′ end using T4 polynucleotide kinase

(Invitrogen). Labeled DNA was separated from unincorporated nucleotide using MicroBioSpin columns (BioRad). Synthetic oligonucleotides were annealed in 10 mM Tris-HCl pH 8.0, 1mM EDTA by heating to 90°C for 3 min in a heat block, followed by slow cooling to room temperature over 3 h. Annealed substrates were further purified by separation on 10% polyacrylamide gels in 90 mM Tris-borate, 2 mM EDTA. A 20 nt fluorescein 3′-end-labeled oligonucleotide (o-FLU$_{20}$) was annealed to o-SS$_{20}$ for fluorescence anisotropy experiments. For FRET DNA bending experiments, o-FLU$_{20}$ was annealed to its complement o-TET$_{20}$. Fluorescein was linked by a C6 alkylamino spacer and tetramethylrhodamine by a C9 alkylamino spacer.

Table S2. Average inter-fluorophore distance following addition of NinH.

A

	average interfluorophore distance, R / Å	change in interfluorophore distance, R / %
no protein	74.74 (±0.06)	0.00 (± 0.08)
BSA	74.47 (±0.08)	-0.36 (± 0.11)
NinH	71.28 (±0.12)	-4.63 (± 0.16)

B

	average interfluorophore distance, R / Å	change in interfluorophore distance, R / %
no protein	70.72 (±0.93)	0.00 (±2.28)
BSA	69.60 (±1.97)	-0.22 (±3.04)
NinH	65.03 (±1.43)	-8.03 (±2.41)

Distances, R, are tabulated with the percentage change relative to the no protein control for both (*A*) steady-state and (*B*) time-resolved FRET experiments. Experiments were performed in triplicate and the standard error of the mean is shown in parenthesis. The results of the steady-state experiments appear to be more precise than those of the time-resolved assay as indicated by the smaller standard error, although it may slightly underestimate the amount of DNA bending. Another factor that may have influenced calculated steady state energy transfer efficiencies is scattered excitation light in the donor fluorescence spectra. Furthermore, the calculated steady state efficiencies will not capture any orientational motion by the donor and acceptor chromophores during their excited state lifetimes. It is not unreasonable to assume that immediately after photoexcitation the mutual orientation of the donor-acceptor pair is more favourable for FRET, which in turn would explain the higher efficiencies obtained in the time-resolved experiments. The effect is a slight underestimation of the calculated steady state efficiencies.

Table S3. Calculation of dsDNA bending by NinH protein.

A

	θ_T (°)	+ 1 SEM	- 1 SEM
BSA	9.8	8.3	11.1
NinH	35.0	34.7	35.2

B

	θ_T (°)	+ 1 SEM	- 1 SEM
BSA	20.4	0.0	34.0
NinH	46.2	39.9	51.8

Duplex DNA bend angles were calculated from steady-state (*A*) and time-resolved (*B*) data. θ_T denotes the angle calculated using a triangular, single-point bend model. Since the bend angle is not linearly dependent on distance, the values for plus and minus one standard error of the mean are not equal and are thus tabulated separately. It was assumed that the DNA construct was approximately straight in the no protein control and the observed interfluorophore distance is in good agreement with a structural model of a straight DNA double helix of 20 bp, with C7 and C9 fluorophore spacers. Without a crystal structure of DNA bound to protein, it is not possible to determine the conformation with more accuracy.

A

CAT ATG ACG TTC TCA GTA AAA ACC ATT CCA GAC ATG CTC GTT GAA ACA TAC GGA AAT CAG ACA GAA GTA GCA CGC AGA CTG AAA TGT AGT CGC GGT ACG GTC AGA AAA TAC GTT GAT GAT AAA GAC GGG AAA ATG CAC GCC ATC GTC AAC GAC GTT CTC ATG GTT CAT CGC GGA TGG AGT GAA AGA GAT GCG CTA TTA CGA AAA AAT TGA TGG CTC GAG

N18A (AAT → GCA)

S29A (AGT → GCA)

R30A (CGC → GCA)

T32A (ACG → GCA)

K35A (AAA → GCA)

S29A R30A (AGT → GCA, CGC → GCA)

H45A (CAC → GCC)

H55A (CAT → GCG)

R56A (CGC → GCC)

H55A R56A (CAT → GCG, CGC → GCC)

ΔC25 (ATG → TAG)

B

GTCGAC*GCAGCTGCAGCTGCG*ATGACGTTCTCAGTAAAAACCATTCCAGACATGCTCGT
TGAAGCATACGGAAATCAGACAGAAGTAGCACGCAGACTGAAATGTAGTCGCGGTACGG
TCAGAAAATACGTTGATGATAAAGACGGGAAAATGCACGCCATCGTCAACGACGTTCTCA
TGGTTCATCGCGGATGGAGTGAAAGAGATGCGCTATTACGAAAAAATTGAGGATCC

Figure S1. Codon optimised constructs for *ninH* and site-directed mutants.

(**A**) Mutated codons are highlighted in blue and their replacements listed below. The *ninH* was synthesized with flanking NdeI and XhoI sites and inserted in pET28a(+). (**B**) Insert containing the *ninH* gene was inserted into pTH6838 (reference 19 in the main text) using SalI and BamHI sites to create a GST-NinH fusion. An Ala_5 linker was introduced between the GST and NinH sequences (residues in italics between the SalI site and the original NinH start codon that comprises 5 codons). Restriction sites used in cloning are underlined. Start and stop codons are labeled in red.

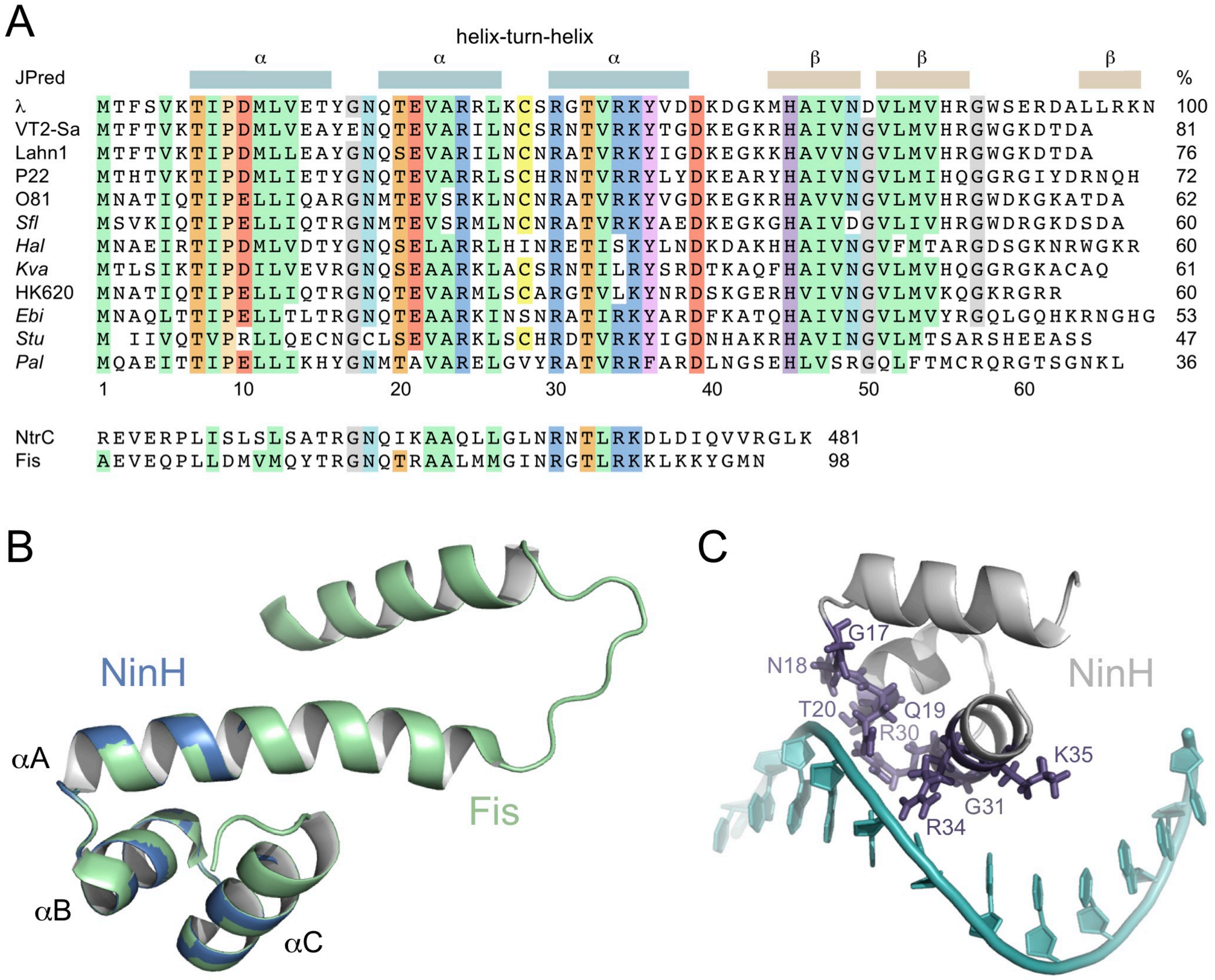

Figure S2. **Structural modeling of NinH and sequence alignment of representative homologues.**

(**A**) The NinH protein from phage λ (P03771) was aligned with homologous representatives from *E. coli* O157:H7 phage VT2-Sakai (P69177; VT2-Sa), *E. coli* O84:H^{-} phage Lahn1 (Q777W6; Lahn1), *Salmonella* phage P22 (Q38669), *E. coli* O81 ED1a (O81; B7MPX0), *Shigella flexneri* 2a prophage (*Sfl*; A0A0C7N204), *Hafnia alvei* (*Hal*; G9Y3Q3), *Klebsiella variicola* At-22 (*Kva*; D3R9E5), enterobacteria phage HK620 (Q9AZ09), *Erwinia billingiae* Eb661 (*Ebi*; D8MV69), *Siccibacter turicensis* DSM 18703 (*Stu*: C9XYF4) and *Providencia alcalifaciens* DSM 30120 (*Pal*; B6XGT3). The percentage identity with λ NinH is shown on the right of each sequence. Conserved residues are highlighted. Secondary elements derived from the JPred4 server and the predicted helix-turn-helix (HTH) motif are indicated above the alignment. The C-terminal HTH of *Azospirillum brasilense* NtrC (P45671) and *E. coli* Fis (P0A6R3) that correspond to the similar region in NinH are shown to facilitate comparisons. (**B**) Superposition of NinH (residues 6-36) modeled by Phyre^2 on the *E. coli* Fis monomer (residues 26-98; PDB 1FIP). NinH is colored blue and Fis pale green. NinH helices are labelled αA, αB, and αC and correspond to αB, αC, and αD in Fis. (**C**) Predicted NinH DNA

ligand binding site from I-TASSER using a model based on the *Drosophila melanogaster* Brinker DNA binding domain (PDB: 2GLO). Residues G17, N18, Q19, T20, 30, 31, 34, 35 (highlighted in purple) are predicted to be involved in contacting the DNA and correspond well to those residues conserved between Fis and NinH (see Figure 1A). The model produced by I-TASSER only generated a single DNA strand of the duplex.

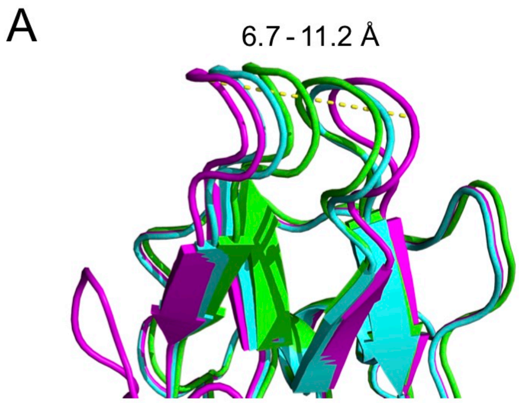

B

	His-NinH
Radius of gyration [Å] - Gunier	21.61
Radius of gyration [Å] – p(r)	21.66
Molecular Weight [kDa]	20.6
MW probability [%]	37
Credibility Interval [kDa]	19.6-21.5
Credibility Interval Probability [%]	95
Maximal diameter [Å]	82.7

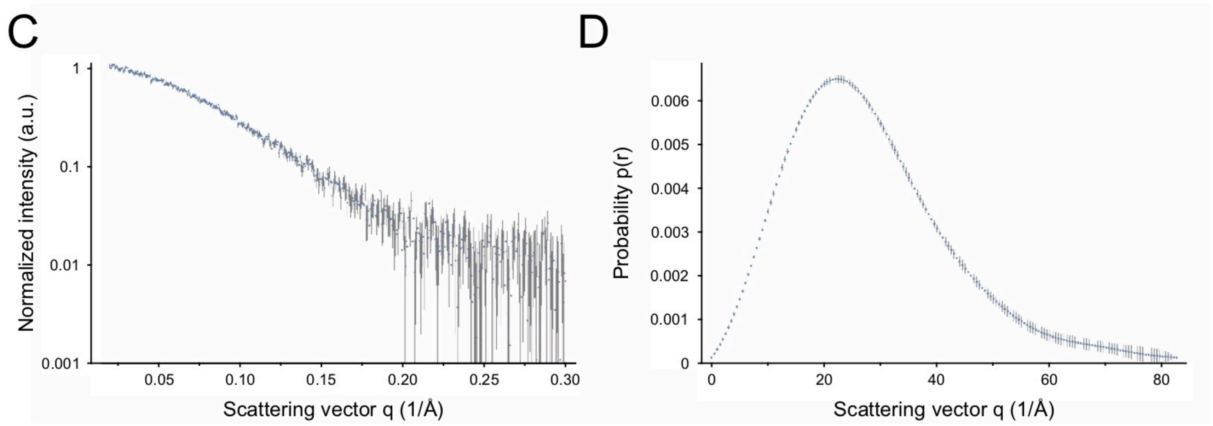

Figure S3. Structural modeling and SAXS analysis of NinH (SASBDB ID: SASDHS4).
(**A**) Comparison of different models from open state (magenta) with a distance of 11.2 Å between C-terminal loops to closed (green) with a distance of 6.7 Å. (**B**) Structural parameters derived from SAXS analysis of His-NinH. (**C**) Scattering profile of His-NinH with measurement errors. (**D**) Real size distribution of His-NinH suggesting a globular object with maximal diameter of 82.7 Å.

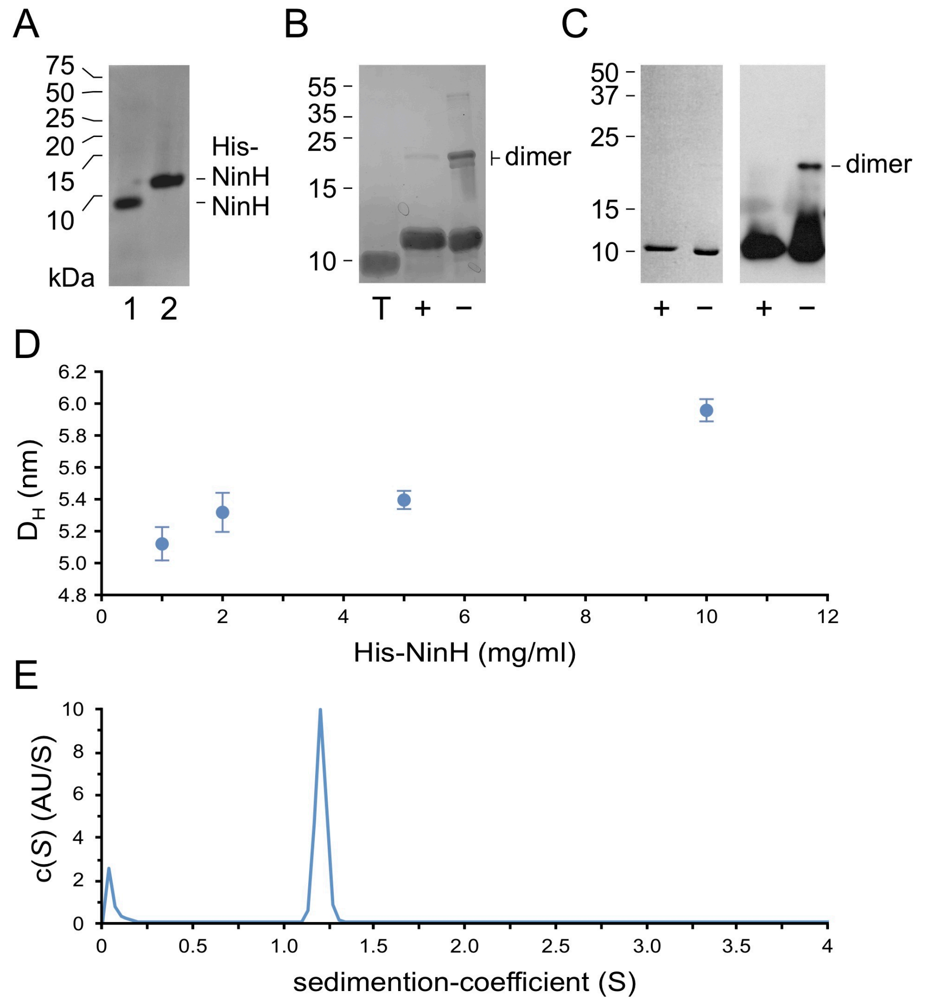

Figure S4. Quaternary analysis of His-NinH.

(**A**) 5 µg of purified NinH and His-NinH separated on a 10-20% gradient Tris-tricine SDS gel. (**B**) 15% SDS-PAGE showing thrombin cleavage of His-NinH to remove the histidine tag and samples of boiled (+) and unboiled (–) His-NinH samples. 2 µM of protein mixed at a 1:1 ratio with thrombin (Sigma) and incubated at room temperature overnight was sufficient to remove the histidine tag. (**C**) 15% SDS-PAGE (left) of boiled (+) and unboiled (–) His-NinH samples and a Western blot (right) probed with anti-polyhistidine antibodies. For blotting, NinH samples were transferred to a PVDF membrane by electroblotting in 10 mM CAPS and 20% (v/v) methanol. Presence of His-NinH protein was detected with monoclonal anti-polyhistidine antibodies and goat anti-mouse IgG peroxidase conjugate (Sigma). Final detection was made by chemiluminescence by exposing the sample to X-ray film. Marker sizes shown on the left of both insets are in kilodaltons. (**D**) Dynamic light scattering analysis of His-NinH. Performed using a Malvern Zetasizer Nano ZSP with a temperature controller. To remove large aggregates prior to measurement, His-NinH samples were centrifuged at 13,400 rpm for 10 min. Experiments were performed at RT with protein concentration in the 1-10 mg range. (**E**) Analytical ultracentrifugation of His-NinH (this trace is also presented in Fig. 6B).

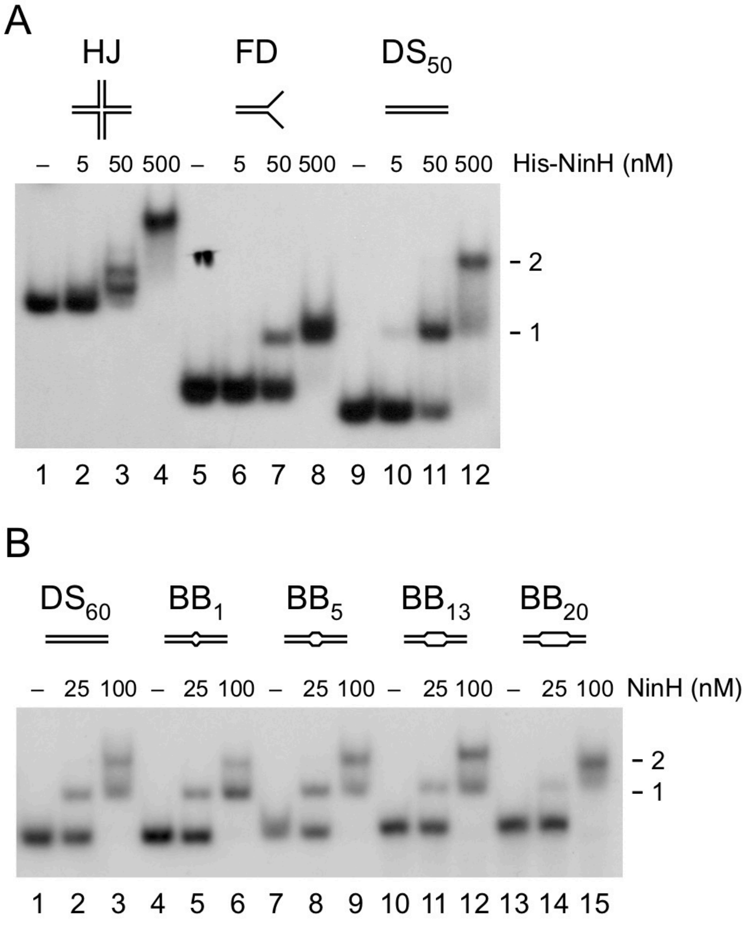

Figure S5. NinH protein DNA binding to branched and bubble DNA structures.

(**A**) Gel mobility shift assay containing 0, 5, 50 and 500 nM His-NinH protein and 0.3 nM of ^{32}P-labeled Holliday junction (HJ; lanes 1-4), forked duplex (FD; lanes 5-8) and a dsDNA (DS_{50}; lanes 9-12) unrelated in sequence to DS_{60}. His-NinH only yielded a single complex on the fork substrate containing a 31 bp stretch of duplex DNA (lanes 6-8), whereas two complexes were noted on the 50 bp duplex, suggesting that there is insufficient space to accomodate two dimers of NinH or that the single-strand flaps interfere with the stable formation of a second complex. Three or four protein-DNA complexes were detected with the Holliday structure consistent with assembly of His-NinH on each arm of the four-way junction (lanes 2-4). (**B**) Wild-type NinH binding to ssDNA, linear dsDNA and bubble DNA. Binding reactions contained 0, 25 and 100 nM NinH protein and 0.15 nM DNA. The gel in (A) was electrophoresed for longer than that in (B) to help separate the multiple complexes observed between His-NinH and the Holliday structure. Dual protein-DNA complexes with similar levels of DNA binding as with DS_{60} were observed in substrates containing centrally-located mismatches of 1, 5, 13 and 20 nt (lanes 4-15) suggesting that NinH simply recognizes the available duplexes within these structures with no enhanced affinity for a single stranded branch or bubble component.

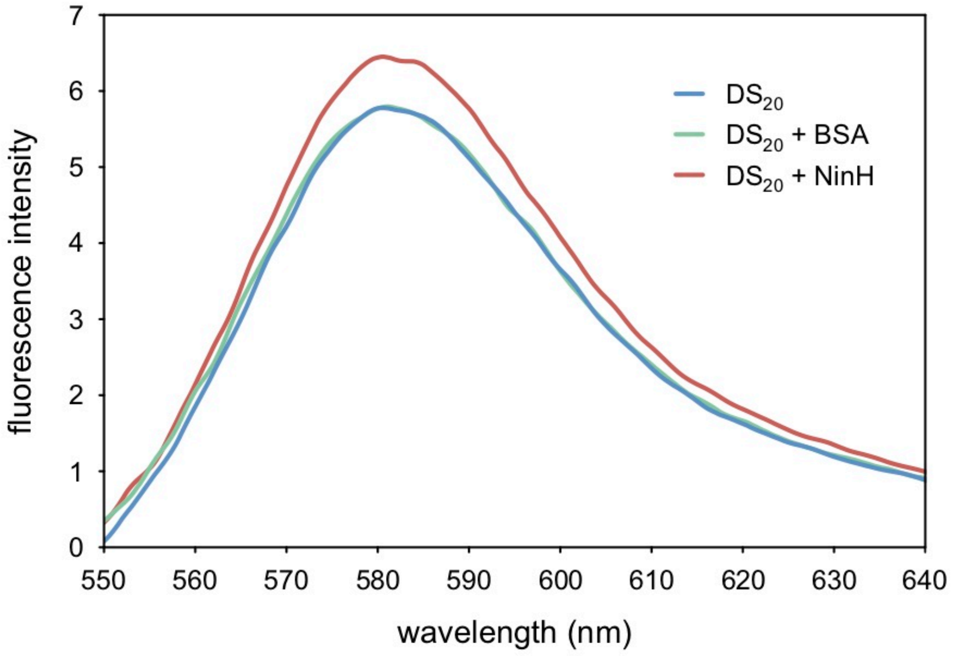

Figure S6. Steady-state FRET analysis of DNA bending induced by NinH.

Fluorescence intensity from donor (fluorescein) to recipient (tetramethylrhodamine) was measured at the emission wavelength for the dual fluorophore-labeled DS_{20} in the absence of protein and with BSA or NinH. The data was used to derive the extent of DNA bending produced by NinH protein.

**E-score scatter plot**	**Z-score scatter plot**
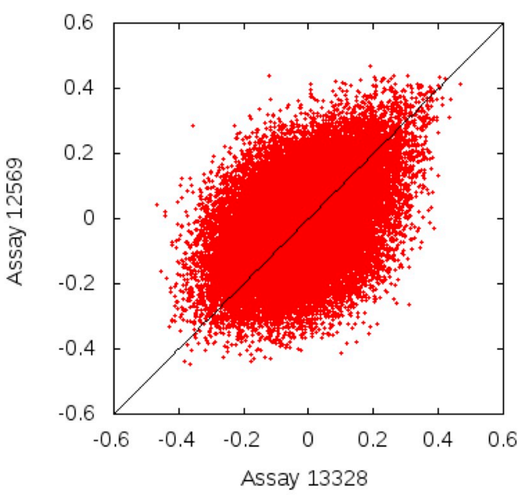	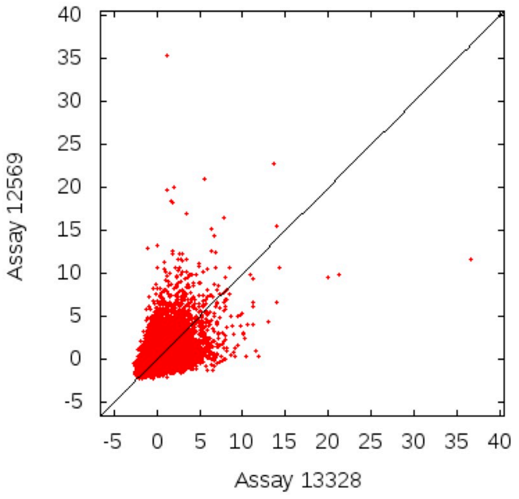
**Correlation Coefficient 0.429521729367**	**Correlation Coefficient 0.4404188519**

Top scoring motifs for Assay 13328
Protein ID: pEX0586.5 **Gene:** NinH **Domain:** HTH **Flag:** Pass_matched_pair **Array:** 1M-ME

8mer E-scores for probeset 'all' (**export E-scores**)

Forward: **Reverse:**

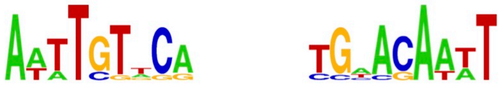

8mer Z-scores for probeset 'all' (**export Z-scores**)

Forward: **Reverse:**

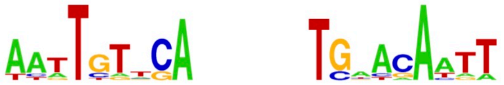

Top 10	Scores	Alignment	Top 10	Scores	Alignment
AAGATTTG	0.46714	CAAATCTT----	AAGATTTG	36.56478	CAAATCTT----
ATTGTTCA	0.43894	--ATTGTTCA--	TTGTACAA	21.22685	---TTGTACAA-
ATTTGTTC	0.43168	-ATTTGTTC---	ATTGTTCA	19.93458	--ATTGTTCA--
AAATCTTG	0.42200	-AAATCTTG---	GGCCAAGA	14.34405	-TCTTGGCC---
CGTCCAAA	0.41242	--TTTGGACG--	TGTGCACA	14.02303	----TGTGCACA
TTGTACAA	0.41222	---TTGTACAA-	AAATCTTG	14.01291	-AAATCTTG---
ATTGTACA	0.40363	--ATTGTACA--	ATTGTACA	13.68501	--ATTGTACA--
TGTGCACA	0.40214	----TGTGCACA	ATTTGTTC	13.00293	-ATTTGTTC---
AATTGTTC	0.39980	-AATTGTTC---	AAGTTATG	11.93817	-AAGTTATG---
ATTTGTGC	0.39864	-ATTTGTGC---	AATTGTTC	11.58018	-AATTGTTC---

Top scoring motifs for Assay 12569
Protein ID: pEX0586.4 **Gene:** NinH **Domain:** HTH **Flag:** Pass_matched_pair **Array:** 1M-HK

8mer E-scores for probeset 'all' (**export E-scores**)

Forward: **Reverse:**

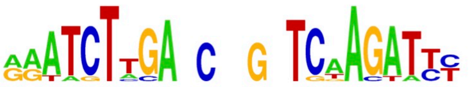

8mer Z-scores for probeset 'all' (**export Z-scores**)

Forward: **Reverse:**

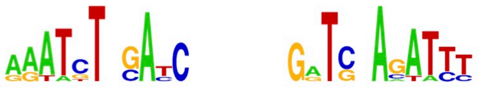

Top 10	Scores	Alignment	Top 10	Scores	Alignment
AAGAAACT	0.46841	AAGAAACT-----	AAGAAACT	35.38191	AAGAAACT-------
AATCTTGA	0.43772	---AATCTTGA--	ATTGTACA	22.75548	---ATTGTACA----
CAAGATCC	0.43759	--GGATCTTG---	TTGATCAA	20.92826	-------TTGATCAA
AGATCTAG	0.43659	--AGATCTAG---	ATTTGCAC	20.01673	----ATTTGCAC---
AGATCTCG	0.43515	--AGATCTCG---	AGATCTCG	19.72582	--AGATCTCG-----
ATTGTACA	0.43324	---ATTGTACA--	AATCTTGA	18.33434	---AATCTTGA----
CAAGATTC	0.43013	--GAATCTTG---	GATCAAGA	18.21591	-----TCTTGATC--
GCTCAAGA	0.42861	-----TCTTGAGC	AGATCTAG	16.94656	--AGATCTAG-----
GATCAAGA	0.42857	-----TCTTGATC	AATGTACA	16.47655	---AATGTACA----
AAATCTTG	0.42780	--AAATCTTG---	AAATCTTG	15.55195	--AAATCTTG-----

Figure S7. PBM analysis data for NinH binding to different microarrays (ME and HK). The scatter plots compare the 8-mer E-scores and Z-scores for ME and HK assays. The top ten enriched 8-mers in ME and HK assays are listed and a graphical position-specific scoring matrix (PSSM) sequence logo represents the preferred binding motif.

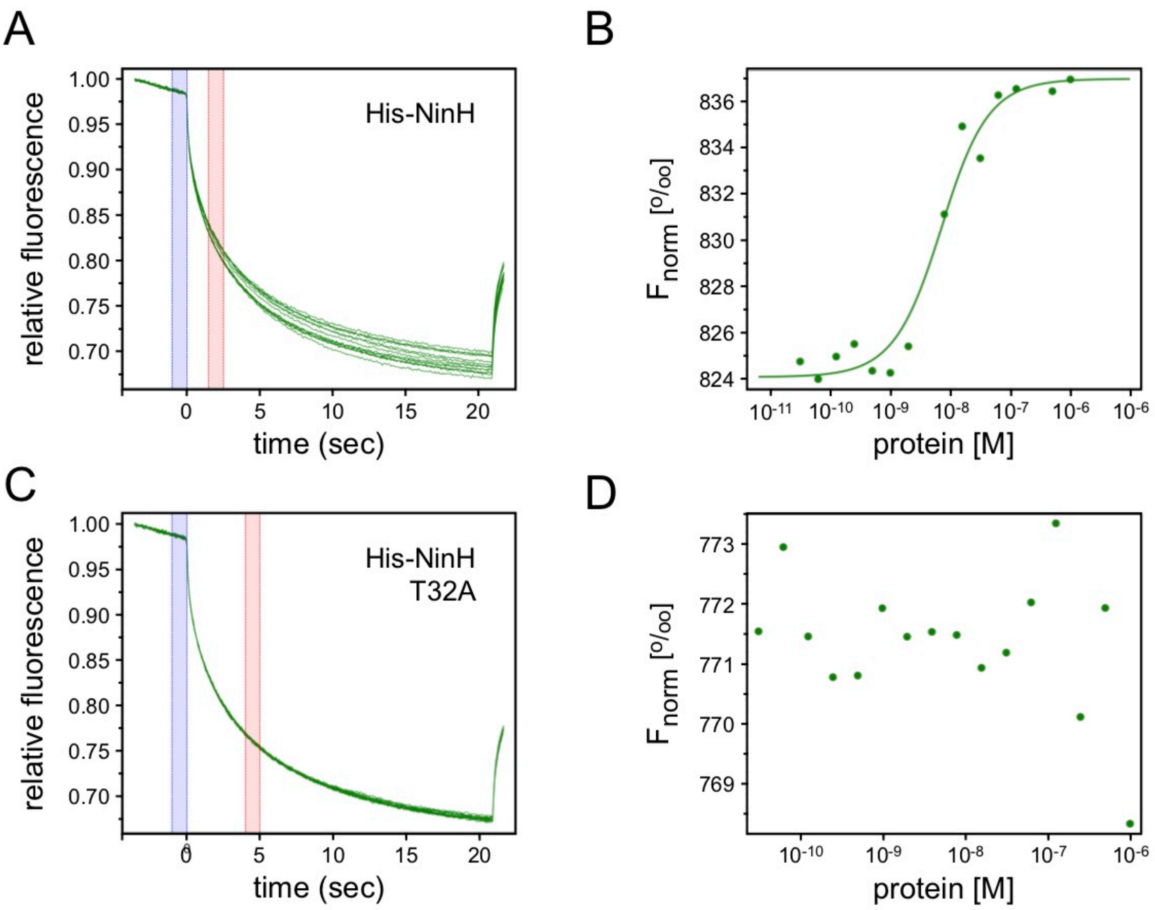

Figure S8. DNA binding of His-NinH and His-NinHT32A to $Cy5_{20}$ dsDNA using microscale thermophoresis (MST).

(**A**) Representative DNA binding trace of 500 nM His-NinH with 2.5 nM $Cy5_{20}$ dsDNA. (**B**) A His-NinH dose response curve was generated. (**C**) Representative DNA binding trace of 500 nM His-NinH T32A with 2.5 nM $Cy5_{20}$. (**D**) A dose response curve could not be generated for His-NinH T32A. Fluorescence was normalized to a starting value of 1 and time spans for F_0 (blue) and F_1 (red) are indicated in A and C. The curve used in B is the same as the His-NinH wt binding isotherm used in Fig. 6D.

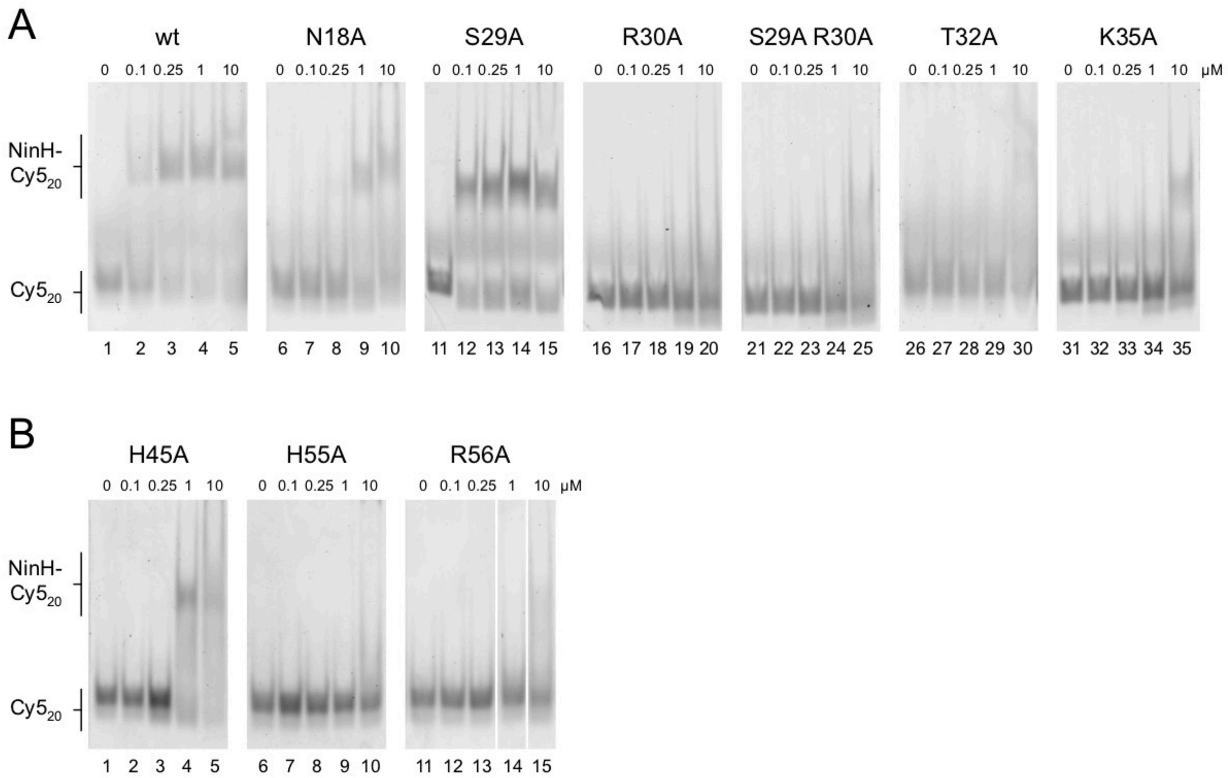

Figure S9. Gel shift assays showing binding of His-NinH wt and mutant proteins to DNA matching the NinH consensus sequence.

(**A**) DNA binding mutants. (**B**) Dimerization mutants. Binding assays were performed with 10 nM 20 bp dsDNA (Cy5$_{20}$) and the indicated amounts of protein in μM. Samples were separated on 7% neutral PAGE gels. Gels for NinH wt and T32A (lanes 1-4 and 26-29) are also shown in Fig. 6C (lanes 1-8) and are reproduced here to facilitate comparisons.

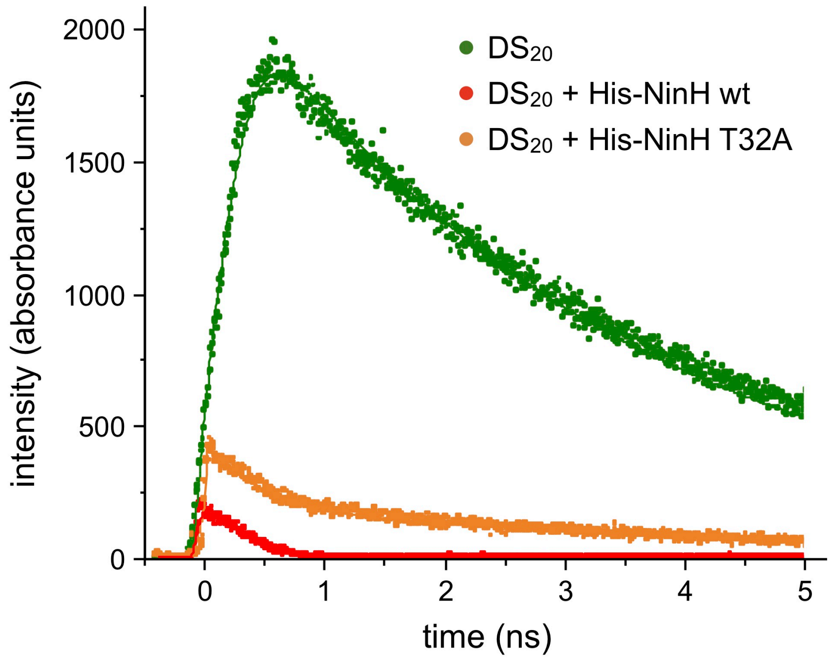

Figure S10. DNA bending by a NinH T32A mutant.

Time-resolved fluorescence spectroscopy of His-NinH wt and His-NinH T32A using a fluorescein-tetramethylrhodamine-labeled DS_{20} substrate DNA. Proteins (1 μM) were incubated with the DNA for 15 min prior to analysis. Fluorescence decay of the donor emission (fluorescein) is normalized with respect to experimental conditions (excitation intensity, acquisition times and sample concentrations). By examining the fluorescence decay of the donor in the donor-acceptor FRET pair (fluorescein-tetramethylrhodamine) of the labeled 20 bp DNA (DS_{20}), qualatitive information on the average donor-acceptor distance was obtained using DNA alone and DNA mixed with His-NinH wt and His-NinH T32A. Calculations were based on the efficiencies of FRET, which in turn were obtained from measured fluorescence lifetimes, as previosuly defined. Here the donor fluorescence lifetime was selected as 4.0 ns which corresponds to isolated fluorescein under similar conditions. For unbound DNA (τ_{DA} = 3.2 ns) a FRET efficiency of 20% was thus obtained. Addition of His-NinH T32A increased the FRET efficiency to 60% indicating some limited DNA binding and bending. However, addition of His-NinH yielded the strongest effect with a FRET efficiency of 99.2%. The results are consistent with significantly reduced NinH DNA binding, and hence bending, as a result of the T32A mutation.

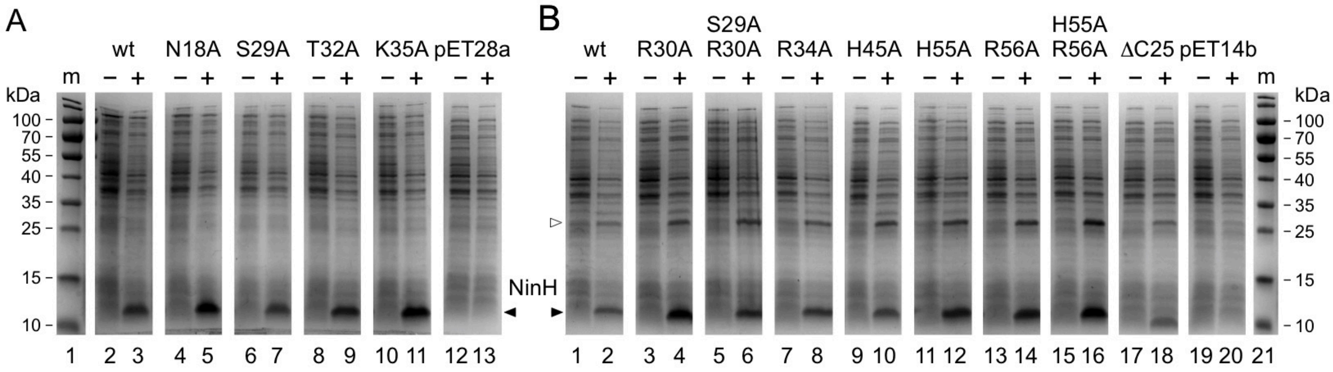

Figure S11. Overexpression of NinH site-directed mutants.

(**A**) NinH clones in pET28a(+). (B) NinH clones in pET14b. BL21-AI carrying pHis-NinH wt, pHis-NinH mutant and vector controls were grown to an A_{650nm} of 0.4 in LB at 37°C. Protein expression was induced in half of each culture by addition of 0.2% arabinose and 1 mM IPTG and growth continued for a further 3 h. Total cellular proteins were separated on 8-16% gradient SDS-PAGE and gels stained with Coomassie blue. Black triangles indicate NinH protein. m = marker. A white triangle in B indicates the position of another induced protein in these samples, potentially the product of the plasmid ampicillin resistance gene, *bla*, which has a molecular mass of 32 kDa. It is unlikely to be a NinH dimer since it is much larger than the 20 kDa expected and a smaller band at this position is not detected in the ΔC25 NinH mutant.
